# Side‐on Coordination in Fluorido Difluoroamido Complexes FMNF_2_ of Heavy Alkaline Earth Metals (M = Ca, Sr, Ba)

**DOI:** 10.1002/chem.202503103

**Published:** 2025-11-05

**Authors:** Xiya Xia, Robert Medel, Sebastian Riedel

**Affiliations:** ^1^ Institut für Chemie und Biochemie – Anorganische Chemie Freie Universität Berlin Fabeckstraße 34/36 14195 Berlin Germany

**Keywords:** alkaline earth metals, IR spectroscopy, non‐VSEPR structure, side‐on coordination

## Abstract

Fluorido difluoroamido complexes F'MNF_2_ of heavy alkaline earth metals (M = Ca, Sr, Ba) were prepared through the reactions of laser‐ablated metal atoms with diluted NF_3_ and isolated in cryogenic neon and argon matrices. They were characterized using Fourier‐transform infrared (FTIR) spectroscopy, ^14/15^NF_3_ isotopic substitution and quantum‐chemical calculations. The species feature a F'^−^ and a so far unknown side‐on coordinated η^3^‐NF_2_^−^ ligand with the F'−M−N angle decreasing from Ca to Ba. Bonding analyses suggest that the interactions between the metal center and the ligands are mainly of electrostatic nature. Nevertheless, the orbital interaction shows that an electron donation from the ligands into the empty *n*s and (*n* – 1)d orbitals of the metal center further contributes to the electronic structure.

## Graphical abstract

Fluorido difluoroamido complexes F'MNF_2_ of heavy alkaline earth metals (M = Ca, Sr, Ba) were prepared through the reactions of laser‐ablated metal atoms with NF_3_ and isolated under cryogenic conditions in neon and argon matrices. The species feature a F'−^−^ and a side‐on coordinated η^3^‐NF_2_^−^ ligand with the F'−M−N angle decreasing from calcium to barium.

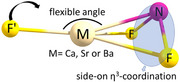

## 1 Introduction

Heavy alkaline earth metal atoms, for example, calcium, strontium and barium, have a fully occupied ns orbital (*n *= 4, 5, 6) but empty (*n* − 1)d orbitals. They tend to lose the two electrons from the ns orbital to form the dication in compounds. However, this simple ionic model cannot explain the fact that most monomeric heavy alkaline earth metal dihalides MX_2_ (M = Ca, Sr, Ba; X = F, Cl, Br, I) have bent instead of linear geometries.^[^ ^1^, ^2^ ^]^ It was concluded that d orbital participation based on increasing relativistic effects is one of the main reasons for the bent structures.^[^ ^3^ ^]^ In line with this perspective, lately, an unusual side‐on coordination mode in alkaline earth metal hypofluorite was observed in our laboratory, with the degree of nonplanarity increasing progressively from calcium to barium.^[^ ^4^ ^]^ Heavy alkaline earth metals can also mimic transition metals in the formation of octahedral carbonyl M(CO)_8_ and nitrogen complexes M(N_2_)_8_, which follow the 18‐electron rule.^[^ ^5^, ^6^ ^]^ Moreover, growing evidence shows that the d orbitals of the heavy alkaline earth metals play an important role in bonding and molecule activation.^[^ ^7^, ^8^, ^9^ ^]^ Based on this assumption, it was suggested treating the heavy alkaline earth metals more as transition metals rather than main‐group elements.^[^ ^10^, ^11^ ^]^

**Scheme 1:**
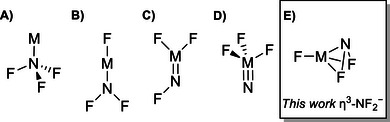
Schematic representation of isomeric NF_3_ metal products: A) nitrogen trifluoride, B) fluorido η^1^‐difluoroamido, C) difluorido fluoroimido, D) trifluorido nitrido, and E) fluorido η^3^‐difluoroamido metal complexes.

Nitrogen trifluoride NF_3_ is a stable gas at room temperature, a potent greenhouse gas and a strong oxidizer at decomposition temperatures.^[^ ^12^ ^]^ Despite a variety of applications of NF_3_ in industry, including, for example, electronics,^[^ ^13^ ^]^ there are still blank spots in the chemistry of this compound. In particular, investigations on metal‐NF_3_ interactions are rather scarce. Thus, the reaction of NF_3_ with group 4, 6, and 8−11 transition metals has been investigated by matrix‐isolation Fourier‐transform infrared (FTIR) spectroscopy in recent years.^[^ ^14^, ^15^, ^16^, ^17^, ^18^, ^19^ ^]^ Depending on the metal used, four isomeric types of products have already been reported (Scheme 1), namely complexes of A) nitrogen trifluoride,^[^ ^14^ ^]^ B) fluorido difluoroamido,^[^ ^15^ ^]^ C) difluorido fluoroimido,^[^ ^16^ ^]^ and D) trifluorido nitrido^[^ ^17^, ^18^, ^19^ ^]^ ligands, all with η^1^‐coordination.

Moreover, theoretical studies on complexes of group 1 and 2 monocations M^+^ (M = H, Li, Na, K, Be, Mg) with nitrogen trifluoride suggested that for M = Li, Na, and K, the ion‐dipole complexes M^+^−NF_3_ could be observed as stable species in the gas phase.^[^ ^20^ ^]^ On the other hand, FM^+^−NF_2_ complexes are more favorable for M = H, Be, and Mg.^[^ ^21^ ^]^ Furthermore, the neutral complexes of magnesium and calcium with (formally) analogous ammine ligands M(NH_3_)*_n_* have also been characterized experimentally and by quantum‐chemical calculations.^[^ ^22^, ^23^ ^]^

However, to the best of our knowledge, there is neither a quantum‐chemical nor experimental investigation on the reactions between heavy alkaline earth metals and NF_3_ published.

Here, we present a so far unknown variant of the insertion product of NF_3_ with metals forming a F'MNF_2_ compound with a side‐on coordinated η^3^‐NF_2_^−^ ligand (Scheme 1 E)). This new class of species were prepared through the reactions of laser‐ablated heavy alkaline earth metal atoms M (M = Ca, Sr, Ba) with NF_3_ under cryogenic conditions in solid argon and neon matrices (for experimental details see Supporting Information). The characterization was based on the recorded infrared spectra in conjunction with quantum‐chemical calculations, and isotopic substitution experiments with ^14/15^NF_3_.

## 2 Results and Discussion

**Figure 1:**
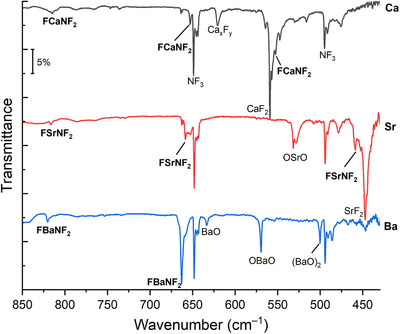
FTIR spectra of different laser‐ablated heavy alkaline earth metal atoms with 0.5% NF_3_ in argon after codeposition at 10 K.

Infrared spectra of the product formation from laser‐ablated metal atoms with 0.5% NF_3_ under excess argon, codeposited at 10 K, are shown in Figure 1.

As a side product, metal fluorides were assigned based on the comparison with the reactions of metal atoms and elemental fluorine (Figures S1 ,  S2). Also, metal oxides were observed in our spectra due to the reactive nature of the heavy alkaline earth metals with oxygen while handling the targets.^[^ ^24^ ^]^ In addition to these absorption bands, previously unreported bands were found in the recorded infrared spectra (Table 1). After codeposition of laser‐ablated calcium atoms with NF_3_ diluted in Ar, new absorptions at 652.7 and 814.8 cm^−1^ are assigned to the symmetric and antisymmetric NF_2_ stretching modes of F'CaNF2. Experiments using isotopically labeled ^15^NF_3_, show corresponding bands which are downshifted to 641.8 and 797.5 cm^−1^, respectively (Figure S3). Furthermore, a new band at 552.2 cm^−1^, overlapping with the antisymmetric mode of CaF_2_, shows the identical behavior as the symmetric and antisymmetric NF_2_ stretching modes upon irradiation (Figures S3 ,  S4). However, it does not show a ^14/15^N isotopic shift, and is therefore assigned to the F'−Ca vibration of F'CaNF_2_. In neon matrices the same product formation was observed with NF_2_ stretching modes slightly red‐shifted to 649.6 and 809.7 cm^−1^ while the F'−Ca vibrational mode was blue‐shifted to 569.3 cm^−1^ (Figure S4).

**Table 1:** Experimental and calculated vibrational wavenumbers ν in cm^−1^, IR intensities in km mol^−1^ (in parentheses) and ^14/15^N isotopic shifts Δν in cm^−1^ for F'MNF_2_ (M = Ca, Sr, Ba) species.^[^ ^a^ ^]^

	exp. in Ar matrices		exp. in Ne matrices		B3LYP^[^ ^b^ ^]^ [harm.]		B3LYP^[^ ^b^ ^]^ [anh.]		CCSD[T]^[^ ^c^ ^]^ [harm.]		
species	ν(^14^N)	Δν(^14/15^N)	ν(^14^N)	Δν(^14/15^N)	ν(^14^N)	Δν(^14/15^N)	ν(^14^N)	Δν(^14/15^N)	ν(^14^N)	Δν(^14/15^N)	assignment mode
F'CaNF_2_	552.2	0.0	569.3	0.0	580.2 (243)	−0.2	575.3 (241)	0.0	583.8	−0.2	ν(F'−Ca)
	652.7	−10.9	649.6	−10.5	640.0 (189)	−11.6	625.7 (170)	−12.3	664.8	−12.2	ν_as_(NF_2_)
	814.8	−17.3	809.7	−16.0	826.5 (50)	−17.7	795.9 (23)	−15.3	827.6	−17.9	ν_s_(NF_2_)
F'SrNF_2_	459.6	0.0	−	−	478.6 (151)	0.0	474.4 (151)	0.0	480.8	0.0	ν(F'−Sr)
	653.9	−12.2	653.4	−11.7	640.8 (182)	−12.0	630.3 (134)	−9.1	666.2	−12.6	ν_as_(NF_2_)
	817.2	−15.7	812.8	−15.9	830.1 (46)	−17.8	832.3 (28)	−15.3	831.1	−17.9	ν_s_(NF_2_)
F'BaNF_2_	663.7	−12.3	662.4	−12.6	657.8 (173)	−12.6	643.2 (170)	−11.7	679.0	−13.1	ν_as_(NF_2_)
	821.1	−15.9	816.1	−15.7	839.4 (44)	−17.8	838.5 (36)	−16.5	837.8	−17.8	ν_s_(NF_2_)

^[a]^ Only wavenumbers above 450 cm^−1^ are listed.

^[b]^ B3LYP/def2‐TZVP.

^[c]^ CCSD(T)/aug‐cc‐pwCVTZ‐(PP).

Laser‐ablation experiments with the next heavier alkaline earth metal, Sr in argon matrices showed the formation of F'SrNF_2_ with product bands at 817.2, 653.9, and 459.6 cm^−1^. The first two bands are assigned to the symmetric and antisymmetric NF_2_ stretching modes with ^14/15^N isotopic shifts of −15.7 and −12.2 cm^−1^, respectively (Figure S5). The band at 459.6 cm^−1^ has virtually no ^14/15^N isotopic shift and is assigned to the F'−Sr vibrational mode. It is observed that the bands of F'SrNF_2_ increase slightly after irradiation with 730 nm. In neon matrices, the symmetric and antisymmetric NF_2_ stretching modes can be observed at 653.4 and 812.8 cm^−1^, respectively (Figure S6). However, the exact position of the F'−Sr vibrational mode of F'SrNF_2_ in neon could not be identified due to overlap with intense strontium fluoride bands and low signal‐to‐noise ratio.

The IR spectra recorded after laser‐ablation of Ba with NF_3_ in solid argon show two new bands at 821.1 and 663.7 cm^−1^, while the band positions shift to 805.2 and 651.4 cm^−1^ upon ^14/15^N isotopic substitution (Figure S7). According to the positions and isotopic shifts these bands are assigned to the symmetric and antisymmetric NF_2_ stretches of F'BaNF2. Subsequent irradiation at 730 nm also slightly increased these bands, supporting their assignment to the same compound. In solid neon they were also observed with small blue shifts to 816.1 and 662.4 cm^−1^ (Figure S8). The F'−Ba stretch is not observed, its predicted position of about 430 cm^−1^ is below the detection limit of the MCT detector of about 450 cm^−1^.

The calculated harmonic and anharmonic vibrational wavenumbers at the B3LYP level as well as the harmonic ones at the CCSD(T) level are shown in Table 1, together with their ^14/15^N isotopic shifts. Complete calculated vibrational data are listed in the Supporting Information. The experimental values are in good agreement with the calculations both at the DFT and CCSD(T) levels, which supports the assignment of the experimentally detected absorptions. Both the symmetric and antisymmetric NF_2_ stretching wavenumbers increase from Ca via Sr to Ba, in qualitative agreement with the calculations. It should be noted that the isotopic shifts of the symmetric NF_2_ vibrational mode tend to be overestimated by about 2 cm^−1^ by the calculations within the harmonic approximation. Taking anharmonicity into account by VPT2 corrections improves the agreement for the symmetric mode, but worsens it for the antisymmetric one.

**Figure 2:**
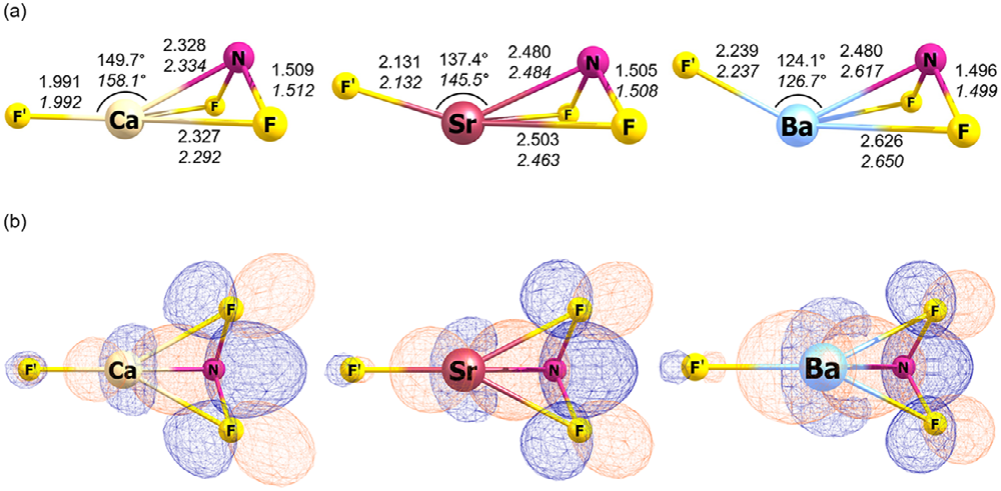
a) Optimized structures of F'MNF_2_ (M = Ca, Sr, Ba) at the B3LYP/def2‐TZVP (normal) and CCSD(T)/aug‐cc‐pwCVTZ(‐PP) (*italics*) levels with bond lengths in Å. b) HOMO of the corresponding molecules at the B3LYP/def2‐TZVP level (isovalue = 0.025 a.u.).

The formation of F'MNF_2_ from the direct insertion of alkaline earth metal atoms into the N─F bond of NF_3_ is calculated to be highly exothermic in the order of 600 kJ mol^−1^ at both B3LYP and CCSD(T) levels of theory (Scheme S1). All F'MNF_2_ species have a ^1^A' ground state with *C*_s_ symmetry. The optimized structures at the B3LYP and CCSD(T) levels are shown in Figure 2, the label F' referring to the fluorido ligand. They all show a NF_2_^−^ ligand side‐on coordinated to the metal center. From calcium to barium, the F−M−N bond angle decreases from about 150° to 120°. No further minimum structure could be located. End‐on coordination, as known for group 11 F'MNF_2_ complexes,^[^ ^7^ ^]^ shows imaginary frequencies (see Supporting Information) and is less stable for the heavy alkaline earth metal complexes by about 74 kJ mol^−1^ when *C*_2v_ symmetry is enforced (Table S1).

The highest occupied molecular orbital (HOMO) shows strong contributions from the π* HOMO of isolated NF_2_^−^ as well as from the vacant d orbitals of the isolated metal. From calcium to barium, the orbital overlap increases, which correlates with a shortening of the N─F bonds. This is in line with the experimental increase of the NF_2_ stretching wavenumbers from calcium to barium.

As listed in Table 2, natural population analysis (NPA) shows that the metal center carries a positive charge of about +1.8 *e*. Conversely, the F'^−^ and NF_2_^−^ ligands have a negative charge of about −0.9 *e* each. In addition, the Mayer bond orders reveal that only the F'─M and N─F interactions have bond orders larger than 0.5. The Wiberg bond orders show that there is almost no covalent bond character between the metal centers and ligands. They indicate that the interactions between the metal center and the ligands within F'MNF_2_ are mainly electrostatic.

**Table 2:** NPA charges, Mayer and Wiberg bond orders for F'MNF_2_ (M = Ca, Sr, Ba) obtained from electronic ground state structures calculated at the B3LYP/def2‐TZVP level of theory. All values in atomic units.

Property	Atom	F'CaNF_2_	F'SrNF_2_	F'BaNF_2_
NPA Charges	F'	−0.90	−0.91	−0.90
	M	1.81	1.83	1.80
	N	−0.10	−0.10	−0.11
	F	−0.40	−0.41	−0.40
Mayer bond orders	F'−M	0.64	0.55	0.59
	M−N	0.21	0.19	0.26
	N−F	0.74	0.74	0.76
	M−F	0.17	0.15	0.12
Wiberg bond orders	F'−M	0.19	0.16	0.20
	M−N	0.11	0.11	0.13
	N−F	0.83	0.83	0.84
	M−F	0.04	0.03	0.03

This is also further supported by the AIM analysis shown in Supporting Information (Tables S3 , S4). The positive values of the Laplacian of the electron density ∇^2^ *ρ*_BCP_ and the small values of the electron localization function (ELF) at the bond critical points between the metal center and the ligands indicate closed shell interactions.

The side‐on coordination may be rationalized by the electrostatic interaction being maximized when the negatively charged fluorine atoms are brought closer to the positively charged metal center. However, the F'−M−N angle decreases from 158.8° for calcium to 125.3° for barium (Figure 2), while closed shell metal ions would not show any preferences for specific coordination geometries. It implies that the orbital interaction within FMNF_2_ is not negligible. In order to get a deeper understanding of the side‐on coordination, the extended transition state–natural orbitals for chemical valence (ETS‐NOCV) analysis was carried out.^[^ ^25^, ^26^, ^27^ ^]^ MF^+^ and NF_2_^−^ were chosen as interacting fragments. The total interaction energy, the sum of the orbital interactions and the pairwise contributions are listed in Table 3.

**Table 3:** Results of the ETS‐NOCV analysis for F'MNF2 (M = Ca, Sr, Ba) using F'M^+^ and NF_2_^−^ as interacting fragments at the B3LYP/def2‐TZVP level. Interaction energies in kJ mol^−1^.

	F'CaNF_2_	F'SrNF_2_	F'BaNF_2_
Δ*E*_tot_	−762.83	−707.62	−651.51
Δ*E*_orb_	−153.13	−142.93	−175.48
Δ*E*_orb(1)_	−61.04	−62.05	−99.04
Δ*E*_orb(2)_	−26.90	−20.84	−18.91
Δ*E*_orb(3)_	−25.19	−23.05	−21.88
Δ*E*_orb(rest)_	−40.00	−36.99	−35.65

**Figure 3:**
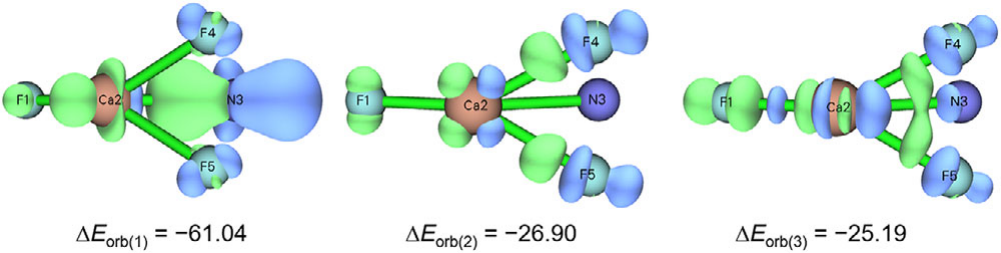
Deformation densities (the electronic charge flow is blue to green) and corresponding Δ*E*_orb_ interaction energies in kJ mol^−1^ from ETS‐NOCV calculations for F'CaNF_2_ performed at B3LYP/def2‐TZVP level for F'Ca^+^ and NF_2_^−^ fragments. The isosurface values is 0.002 au.

The orbital interaction makes only a small contribution to the total interaction energy, consistent with the bonding analyses presented earlier. It stabilizes the compounds by −153.13, −142.93, and −175.48 kJ mol^−1^ for F'CaNF_2_, F'SrNF_2_, and F'BaNF_2_, respectively. It can be concluded that F'BaNF_2_ has stronger orbital interactions than F'CaNF_2_ and F'SrNF_2_. The data also suggest that there are three relatively strong pairwise orbital interactions Δ*E*_orb(1)_−Δ*E*_orb(3)_. The corresponding deformation densities are shown in Figures 3 and S11 for F'CaNF_2_ and Figures S12 − S13 for F'SrNF_2_ and F'BaNF_2_.

It reveals that the orbital interactions mainly come from the donation of the occupied orbitals of the NF_2_^−^ ligand into the vacant *n*s and (*n* – 1)d orbitals of the metal center. Moreover, Δ*E*_orb(1)_ mainly indicates the donation from the π* HOMO of NF_2_^−^ to the metal. From calcium to barium, the interaction energy Δ*E*_orb(1)_ slightly increases, which agrees well with the increasing contribution of the metal to the HOMO, see Figure 2b.

## 3 Conclusion

In conclusion, fluorido difluoroamido complexes F'MNF_2_ were formed through the reaction of laser‐ablated heavy alkaline earth metal atoms with diluted NF_3_ gas under cryogenic conditions in both neon and argon matrices. They were characterized using matrix‐isolation infrared spectroscopy and quantum‐chemical calculations at the B3LYP and CCSD(T) levels and further verified by ^14/15^NF_3_ isotopic substitution. The calculations showed that F'MNF_2_ features a singlet ground state at *C*_s_ symmetry with a M^2+^ center, a coordinated F'^−^ ion and a side‐on coordinated η^3^‐NF_2_^−^ ligand. This is unlike any other metal nitrogen trifluoride product previously described (Scheme 1). It is shown that from calcium to barium, the F'−M−N angle decreases. This observation parallels the non‐VSEPR structures of heavy alkaline earth metal difluorides MF_2_, in which the F−M−F angle decreases down the group.^[^ ^28^ ^]^ This observation is also comparable to the side‐on coordinated alkaline earth metal hypofluorites FM(η^2^‐OF), where the non‐planarity increases from calcium to barium.^[^ ^4^ ^]^ It further shows that for heavy alkaline earth metals, the change of geometry down the group applies not only for homoleptic heavy alkaline earth metal complexes, but also for heteroleptic ones. Furthermore, a side‐on coordination is preferred for polyatomic ligands in molecular alkaline earth metal compounds. This uncommon bonding situation was investigated by different bonding analyses. The results suggest that the bonding interactions are mainly of electrostatic nature between the M^2^^+^ metal center and NF_2_^−^ as well as F'^−^ ligands, which is probably the main reason for the side‐on coordination of NF_2_^−^. Moreover, some orbital interactions from the donation of occupied MOs of the ligands into the vacant *n*s and (*n* – 1)d orbitals of the metal center affects the F'−M−N angle and N─F bond length within the FMNF_2_. This result further supports that the heavy alkaline earth metals have some transition metal character and their empty (*n* – 1)d orbitals play a crucial role in the nature of the chemical bonding of their compounds and have a significant influence on the formed structure.

## Supporting Information

The authors have cited additional references within the Supporting Information.^[^ ^30^, ^31^, ^32^, ^33^, ^34^, ^35^, ^36^, ^37^, ^38^, ^39^, ^40^, ^41^, ^42^, ^43^, ^44^, ^45^, ^46^, ^47^, ^48^ ^]^

## Conflict of Interest

The authors declare no conflict of interest.

## Supporting information



Supporting Information

## Data Availability

The data that support the findings of this study are available in the supplementary material of this article.
