# Supplementary material for: Side‐on Coordination in Fluorido Difluoroamido Complexes FMNF2 of Heavy Alkaline Earth Metals (M = Ca, Sr, Ba)
Source: Chemistry. 2025 Nov 5;31(69):e03103. doi: 10.1002/chem.202503103 (PMC12699167; doi:10.1002/chem.202503103)
Supplement: Supplementary file 1 — Supporting Information [file CHEM-31-e03103-s001.docx]

Supporting Information
©Wiley-VCH 2021
69451 Weinheim, Germany

Side-on Coordination in Fluorido Difluoroamido Complexes FMNF_2_ of Heavy Alkaline Earth Metals (M = Ca, Sr, Ba)

Xiya Xia, Robert Medel, Sebastian Riedel*

**Abstract:** Fluorido difluoroamido complexes F'MNF_2_ of heavy alkaline earth metals (M = Ca, Sr, Ba) were prepared through the reactions of laser-ablated metal atoms with diluted NF_3_ and isolated in cryogenic neon and argon matrices. They were characterized using Fourier-transform infrared (FTIR) spectroscopy, ^14/15^NF_3_ isotopic substitution and quantum-chemical calculations. The species feature a F'^−^ and a so far unknown side-on coordinated η^3^-NF_2_^−^ ligand with the F'−M−N angle decreasing from Ca to Ba. Bonding analyses suggest that the interactions between the metal center and the ligands are mainly of electrostatic nature. Nevertheless, the orbital interaction shows that an electron donation from the ligands into the empty *n*s and (*n*–1)d orbitals of the metal center further contributes to the electronic structure.

Table of Contents

[Experimental Procedures 3](#_Toc161674930)

[Results and Discussion 4](#_Toc161674931)

[IR spectra 4](#_Toc161674932)

Calculated reaction energies [8](#_Toc161674933)

[Energy scan of F'MNF_2_ by changing the F'−M−N angle 9](#_Toc161674934)

[ETS-NOCV analysis 10](#_Toc161674935)

[Comparison of different isomers of F'MNF2 21](#_Toc161674936)

[QTAIM analysis data 22](#_Toc161674937)

[Calculated molecular structures and vibrational data 23](#_Toc161674938)

[References 35](#_Toc161674939)

[Author Contributions 35](#_Toc161674940)

Experimental Procedures

Fluorido difluoroamido complexes FMNF_2_ of heavy alkaline earth metals (M = Ca, Sr, Ba) were prepared by the co-deposition of laser-ablated metal atoms with ^14/15^NF_3_ diluted in neon (99.999% purity, Air Liquide) or argon (99.999% purity, Sauerstoffwerk Friedrichshafen). The gases were mixed in a stainless-steel mixing chamber equipped with a manometer. The mixing chamber was connected to the self-built matrix chamber by a stainless-steel capillary. The deposition process took place onto a gold-plated copper mirror. FTIR spectra were recorded on a Bruker Vertex 80v spectrometer using a liquid-nitrogen-cooled MCT (mercury cadmium telluride) detector with a resolution of 0.5 cm^−1^ in the range of 4000−450 cm^−1^. The FTIR spectra after co-deposition of laser-ablated heavy alkaline earth metals with diluted F_2_ were recorded on a similar apparatus but with a Bruker Vertex 70 spectrometer and a CsI window in transmission mode. To maintain cryogenic temperatures, a closed-cycle helium cryostat (Sumitomo Heavy Industries, RDK-205D) was employed within the matrix chamber. Laser ablation was carried out by using a Continuum Inc. Minilite II Nd:YAG laser (*λ* = 1064 nm) with a repetition rate of 10 Hz, a pulse length of 10 ns, a variable pulse energy from 50−60 mJ. The beam was focused onto the target using a Thorlabs N-BK7 (LA1986-C) plano-convex lens with a focal distance of 125.0 mm. The matrix samples were annealed at different temperatures and irradiated by LED lights with different wavelength (730 nm, 625 nm, 528 nm, 470 nm, 365 nm 273 nm) as well as by a full arc mercury lamp (λ > 220 nm).

Density functional theory (DFT) calculations were conducted using the Gaussian16 Revision A.03 program package for initial structural optimization, analysis of harmonic and anharmonic vibrational frequencies, as well relaxed scans along the F'−M−N angle.^[1]^ The hybrid functional B3LYP,^[2–7]^ coupled with the def2-TZVP basis set,^[8,9]^ was employed for these calculations. Furthermore, Natural Bond Orbital (NBO) analysis was performed using NBO 7.0.^[10]^ QTAIM analysis and ETS-NOCV were carried out by Multiwfn Version 3.8.^[11]^

Additionally, CCSD(T) reoptimizations with harmonic frequency calculations were carried out using the Molpro 2021.3 software.^[12–14]^ The augmented triple-ζ basis sets aug-cc-pVTZ were used for nitrogen, oxygen,^[15–17]^ and the aug-cc-pwCVTZ-PP valence basis and associated scalar-relativistic pseudopotential (PP) for calcium, strontium and barium.^[18,19]^

Results and Discussion


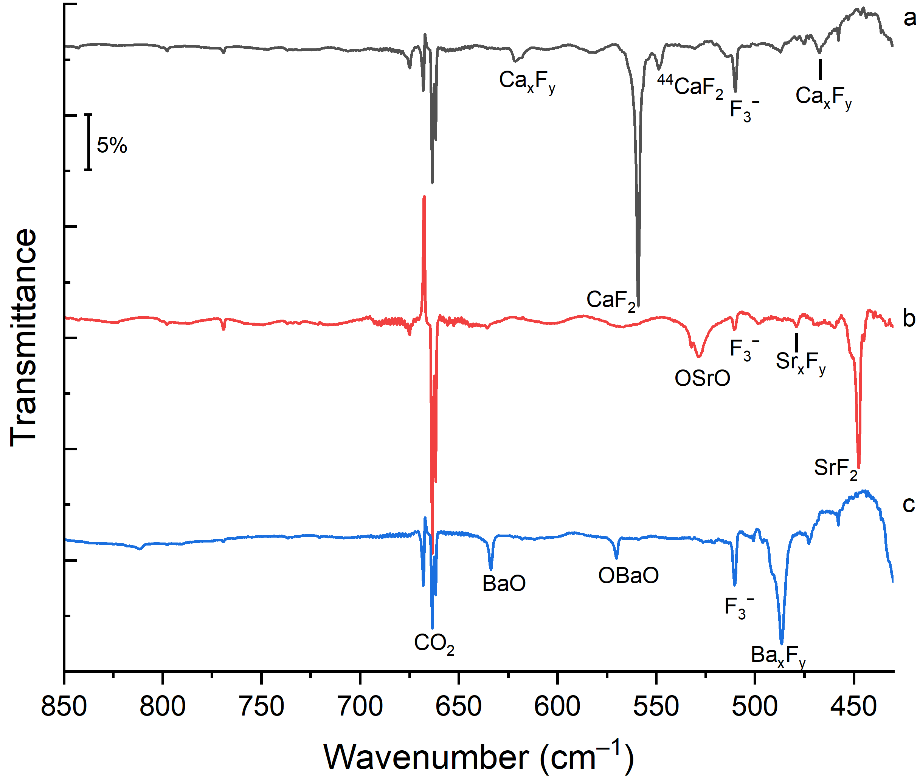
IR spectra

**Figure S1.** IR spectra in argon matrices: (a) after co-deposition of laser-ablated Ca atoms with 0.5% F_2_; (b) after co-deposition of laser-ablated Sr atoms with 0.5% F_2_; (c) after co-deposition of laser-ablated Ba atoms with 0.5% F_2_.


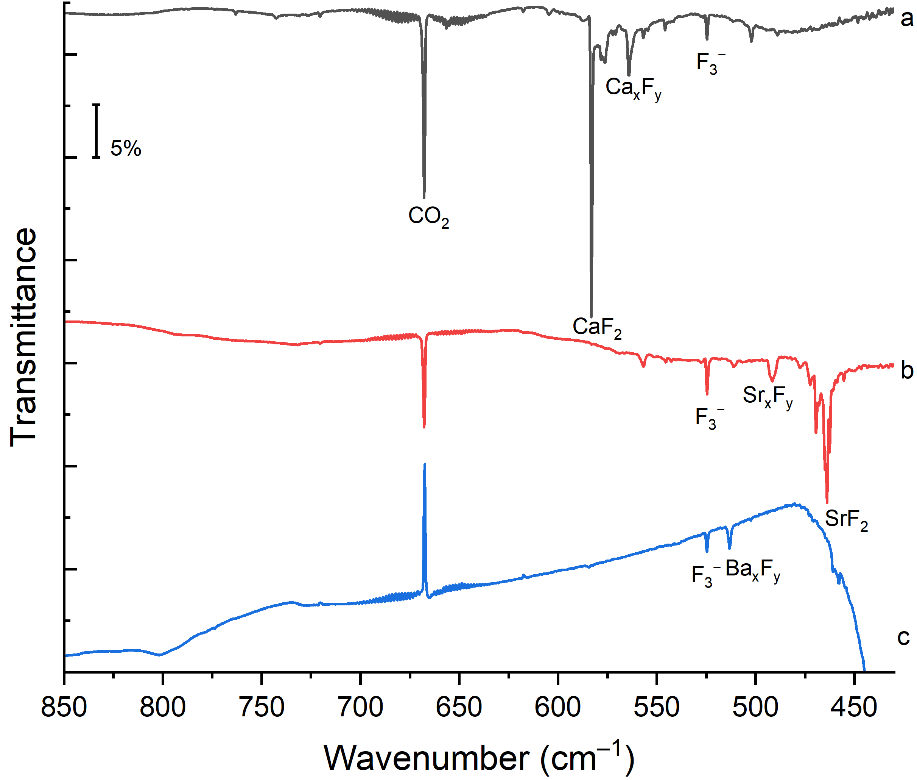


**Figure S2.** IR spectra in neon matrices: (a) after co-deposition of laser-ablated Ca atoms with 0.1% F_2_; (b) after co-deposition of laser-ablated Sr atoms with 0.1% F_2_; (c) after co-deposition of laser-ablated Ba atoms with 0.1% F_2_.


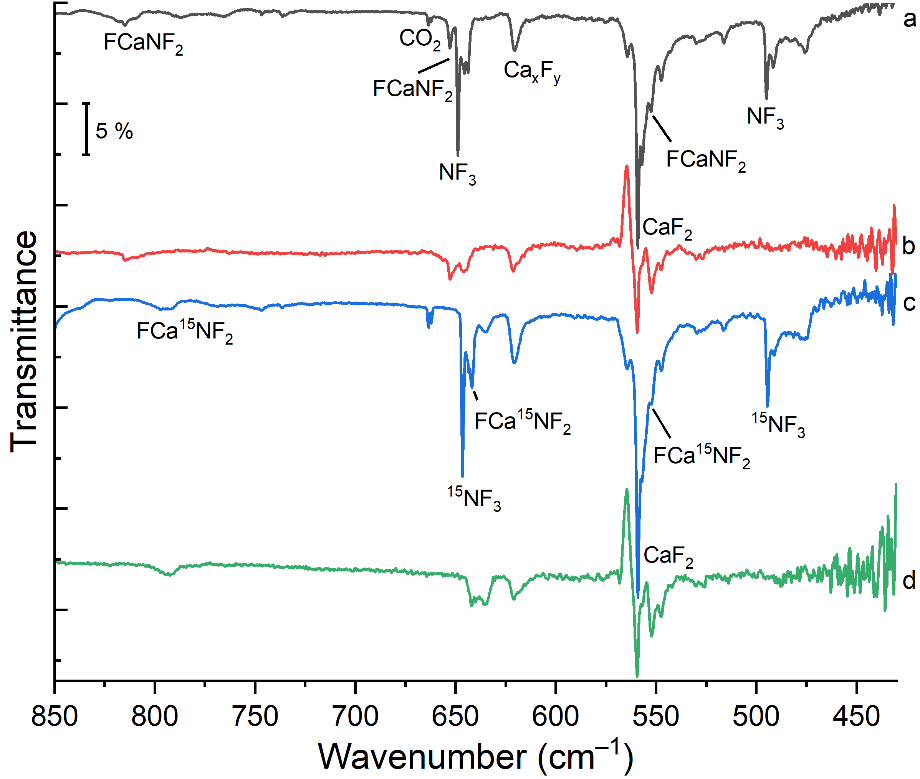


**Figure S3.** IR spectra in argon matrices at 10 K: (a) IR spectrum of reaction products of laser-ablated Ca atoms with 0.5% NF_3_; (b) difference spectrum after irradiation with 625 nm for 10 min; (c) IR spectrum of reaction products of laser-ablated Ca atoms with 0.5% ^15^NF_3_; (d) difference spectrum after irradiation with 625 nm for 10 min.


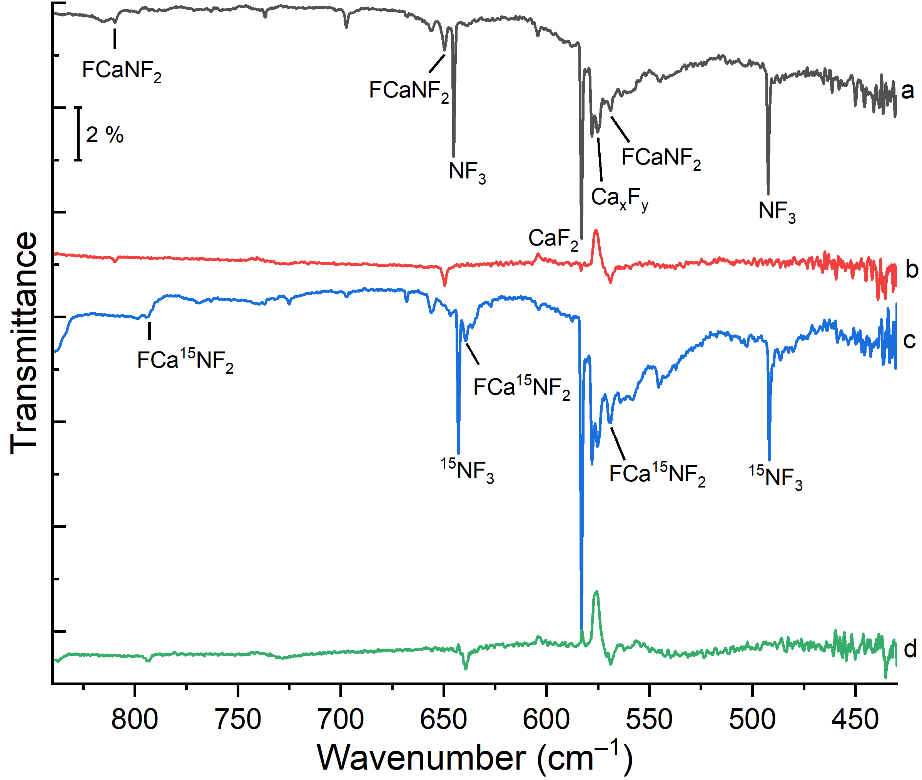


**Figure S4.** IR spectra in neon matrices at 5 K: (a) IR spectrum of reaction products of laser-ablated Ca atoms with 0.1% NF_3_; (b) difference spectrum after irradiation with 528 nm for 10 min; (c) IR spectrum of reaction products of laser-ablated Ca atoms with 0.1% ^15^NF_3_; (d) difference spectrum after irradiation with 528 nm for 10 min.


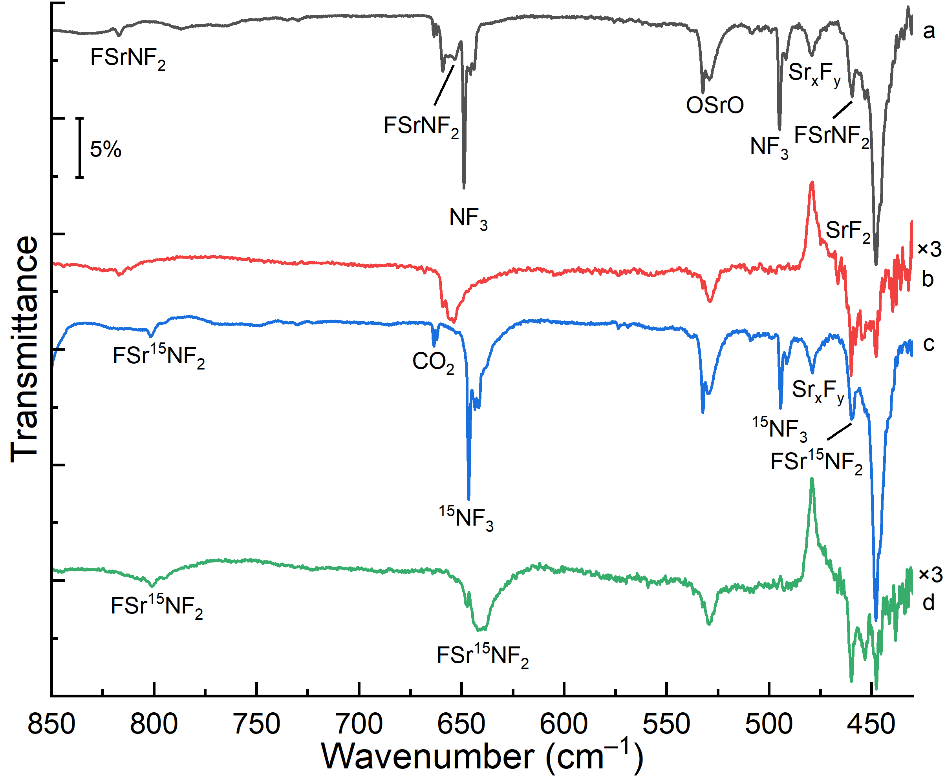


**Figure S5.** IR spectra in argon matrices at 10 K: (a) IR spectrum of reaction products of laser-ablated Sr atoms with 0.5% NF_3_; (b) difference spectrum after irradiation with 730 nm for 10 min; (c) IR spectrum of reaction products of laser-ablated Sr atoms with 0.5% ^15^NF_3_; (d) difference spectrum after irradiation with 730 nm for 10 min.


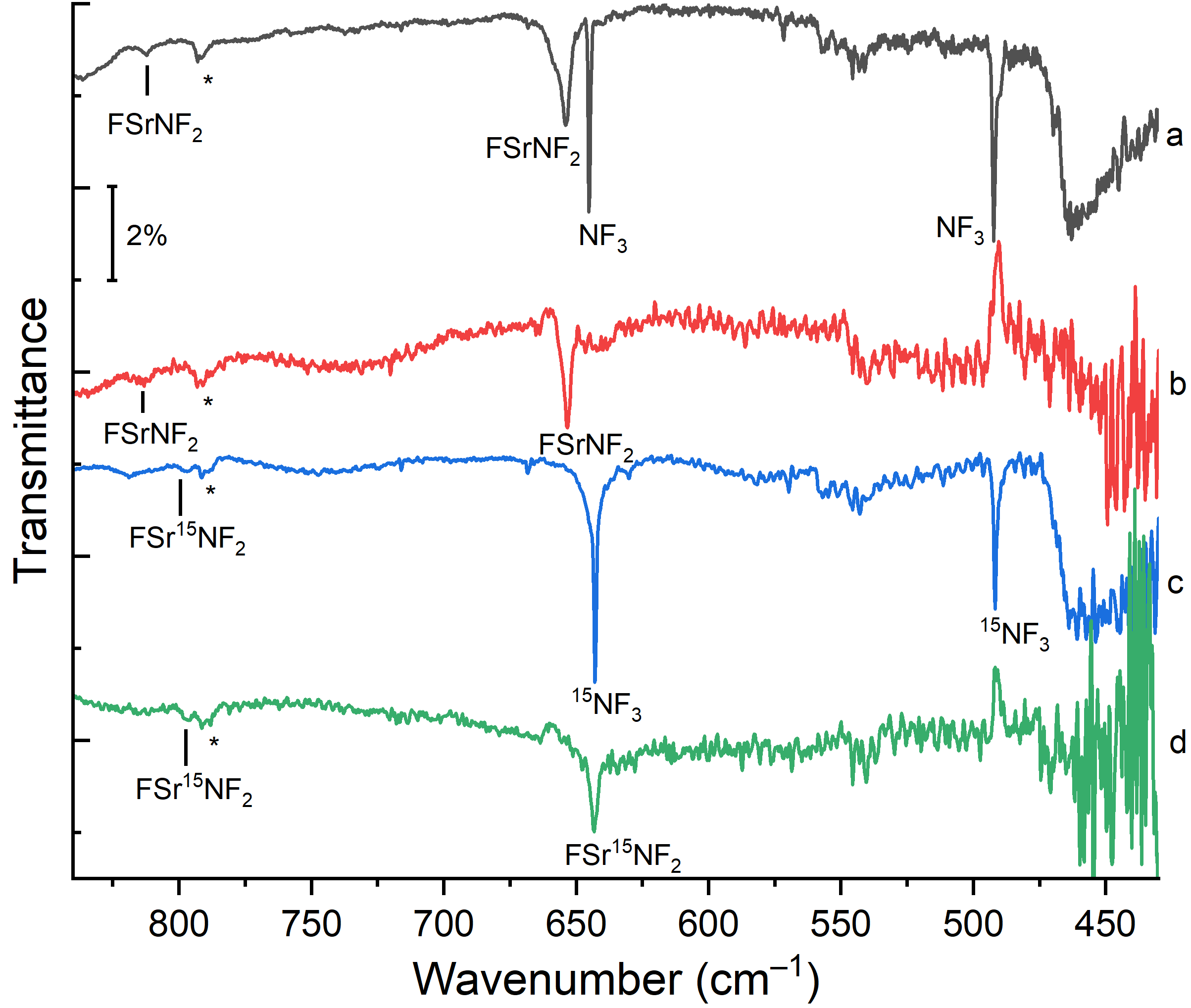


**Figure S6.** IR spectra in neon matrices at 5 K: (a) IR spectrum of reaction products of laser-ablated Sr atoms with 0.1% NF_3_; (b) difference spectrum after irradiation with 625 nm for 10 min; (c) IR spectrum of reaction products of laser-ablated Sr atoms with 0.1% ^15^NF_3_; (d) difference spectrum after irradiation with 625 nm for 10 min. Metal-independent impurities are marked with asterisks.


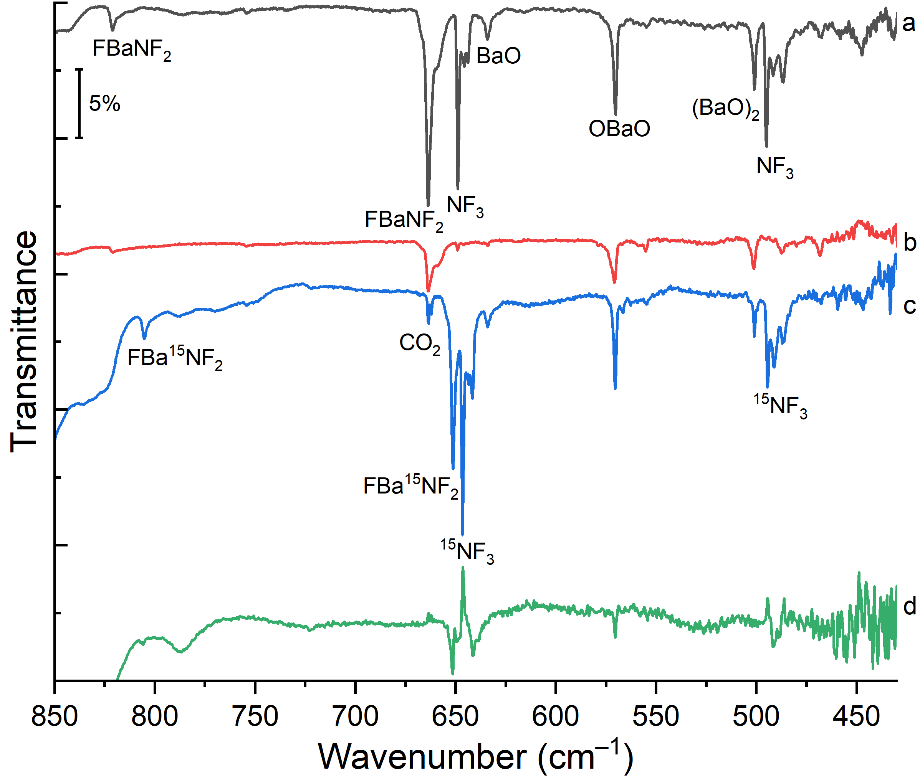


**Figure S7.** IR spectra in argon matrices: (a) IR spectrum of reaction products of laser-ablated Ba atoms with 0.5% NF_3_ at 10 K; (b) difference spectrum after irradiation with 730 nm for 10 min; (c) IR spectrum of reaction products of laser-ablated Ba atoms with 0.5% ^15^NF_3_ at 10 K; (d) difference spectrum after irradiation with 730 nm for 10 min.


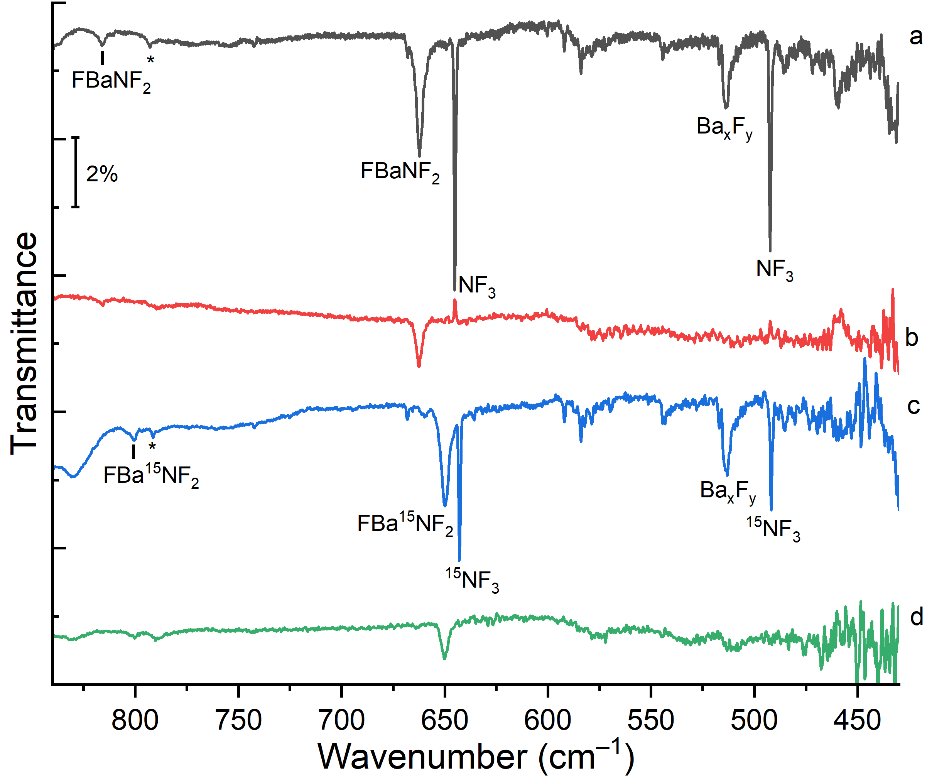


**Figure S8.** IR spectra in neon matrices at 5 K: (a) IR spectrum of reaction products of laser-ablated Ba atoms with 0.1% NF_3_; (b) difference spectrum after irradiation with 730 nm for 10 min; (c) IR spectrum of reaction products of laser-ablated Ba atoms with 0.1% ^15^NF_3_; (d) difference spectrum after irradiation with 730 nm for 10 min. Metal-independent impurities are marked with asterisks.

Calculated reaction energies

**Scheme S1**. Reaction energy of formation of F'MNF_2_ from metal atoms and NF_3_, calculated at B3LYP-D3/def2-TZVP or CCSD(T)/awCVTZ-PP level.

Molecular orbitals (MOs) of NF_2_^−^


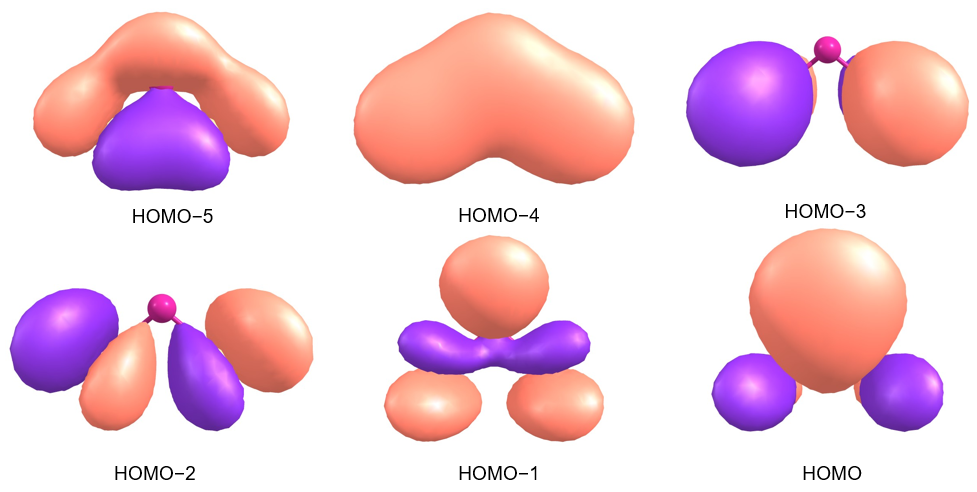


**Figure S9.** Selected MOs of NF_2_^−^ calculated at the B3LYP/def2-TZVP level. Isosurface value = 0.03 a.u.

Relaxed energy scans of F'MNF_2_ along the F'−M−N angle


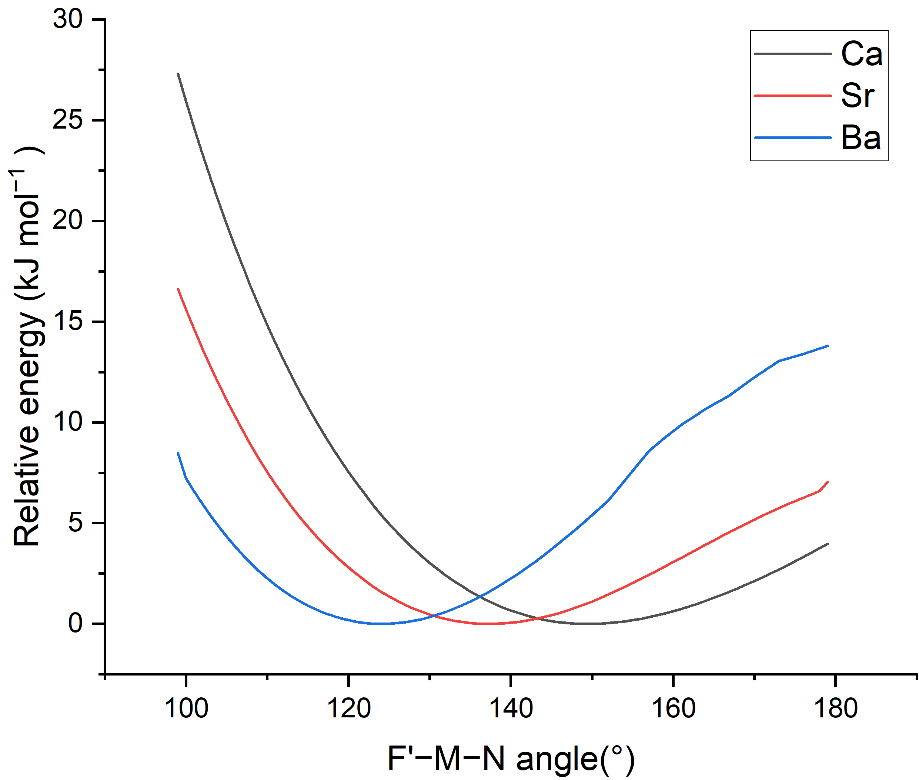


**Figure S10.** Relaxed energy scans of F'MNF_2_ along the F'−M−N angle (M = Ca, Sr, Ba) at B3LYP/def2-TZVP level.

ETS-NOCV analysis

**
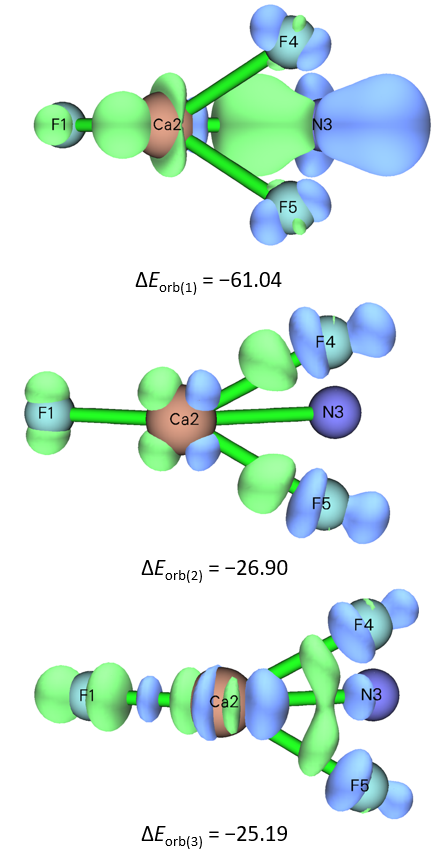
**

**Figure S11.** Deformation densities (the electronic charge flow is blue to green) and corresponding Δ*E*_orb_ interaction energies in kJ mol^−1^ from ETS-NOCV calculations for F'CaNF_2_ performed at B3LYP/def2-TZVP level for F'Ca^+^ and NF_2_^−^ fragments. The isosurface values is 0.002 au. The orbital interaction energies Δ*E* are given in kJ mol^−1^.

Contribution of each basis function shell to NOCV pair/orbitals:

Shell Type Atom Orb. 1 Orb. 160 Pair 1

6 P 1(F ) 0.60 % 0.00 % 0.21 %

16 S 2(Ca) 1.05 % 0.65 % 0.14 %

19 P 2(Ca) 1.41 % 0.15 % 0.45 %

21 P 2(Ca) 3.53 % 2.52 % 0.36 %

23 D 2(Ca) 6.14 % 4.91 % 0.44 %

24 D 2(Ca) 11.08 % 8.96 % 0.76 %

25 D 2(Ca) 0.91 % 0.69 % 0.08 %

29 S 3(N ) 0.93 % 1.98 % -0.38 %

30 S 3(N ) 33.03 % 23.12 % 3.56 %

31 P 3(N ) 4.74 % 9.92 % -1.86 %

32 P 3(N ) 6.87 % 3.48 % 1.22 %

33 P 3(N ) 9.40 % 22.55 % -4.73 %

41 S 4(F ) 6.17 % 4.02 % 0.77 %

42 P 4(F ) 0.47 % 1.32 % -0.31 %

43 P 4(F ) 0.47 % 0.52 % -0.02 %

44 P 4(F ) 1.84 % 3.71 % -0.67 %

52 S 5(F ) 6.17 % 4.02 % 0.77 %

53 P 5(F ) 0.47 % 1.32 % -0.31 %

54 P 5(F ) 0.47 % 0.52 % -0.02 %

55 P 5(F ) 1.84 % 3.71 % -0.67 %

Contribution of various types of shells to NOCV pair/orbitals:

Type Orb. 1 Orb. 160 Pair 1

s: 48.34 % 35.16 % 4.73 %

p: 33.08 % 49.97 % -6.07 %

d: 18.58 % 14.86 % 1.34 %

f: 0.00 % 0.01 % -0.00 %

g: 0.00 % 0.00 % 0.00 %

h: 0.00 % 0.00 % 0.00 %

Contribution of each atom to NOCV pair/orbitals:

Atom Orb. 1 Orb. 160 Pair 1

1(F ): 1.59 % 0.17 % 0.51 %

2(Ca): 24.42 % 18.32 % 2.19 %

3(N ): 55.38 % 61.62 % -2.24 %

4(F ): 9.31 % 9.94 % -0.23 %

5(F ): 9.31 % 9.94 % -0.23 %

Contribution of each basis function shell to NOCV pair/orbitals:

Shell Type Atom Orb. 2 Orb. 159 Pair 2

6 P 1(F ) 3.41 % 0.79 % 0.49 %

7 P 1(F ) 1.95 % 0.60 % 0.25 %

8 P 1(F ) 2.83 % 0.00 % 0.53 %

18 P 2(Ca) 0.66 % 0.01 % 0.12 %

19 P 2(Ca) 6.10 % 0.00 % 1.14 %

20 P 2(Ca) 0.19 % 0.95 % -0.14 %

21 P 2(Ca) 2.34 % 1.26 % 0.20 %

23 D 2(Ca) 10.35 % 10.00 % 0.06 %

24 D 2(Ca) 23.25 % 22.64 % 0.12 %

32 P 3(N ) 0.75 % 0.81 % -0.01 %

40 S 4(F ) 5.10 % 6.42 % -0.25 %

41 S 4(F ) 9.25 % 5.84 % 0.64 %

42 P 4(F ) 3.76 % 8.66 % -0.92 %

43 P 4(F ) 4.30 % 3.27 % 0.19 %

44 P 4(F ) 1.13 % 6.47 % -1.00 %

51 S 5(F ) 5.10 % 6.42 % -0.25 %

52 S 5(F ) 9.25 % 5.84 % 0.64 %

53 P 5(F ) 3.76 % 8.66 % -0.92 %

54 P 5(F ) 4.30 % 3.27 % 0.19 %

55 P 5(F ) 1.13 % 6.47 % -1.00 %

Contribution of various types of shells to NOCV pair/orbitals:

Type Orb. 2 Orb. 159 Pair 2

s: 28.78 % 25.35 % 0.64 %

p: 36.60 % 41.27 % -0.87 %

d: 34.61 % 33.37 % 0.23 %

f: 0.01 % 0.01 % 0.00 %

g: 0.00 % 0.00 % 0.00 %

h: 0.00 % 0.00 % 0.00 %

Contribution of each atom to NOCV pair/orbitals:

Atom Orb. 2 Orb. 159 Pair 2

1(F ): 8.19 % 1.40 % 1.27 %

2(Ca): 42.93 % 34.88 % 1.51 %

3(N ): 0.87 % 0.92 % -0.01 %

4(F ): 24.01 % 31.40 % -1.38 %

5(F ): 24.01 % 31.40 % -1.38 %

Contribution of each basis function shell to NOCV pair/orbitals:

Shell Type Atom Orb. 3 Orb. 158 Pair 3

5 S 1(F ) 1.76 % 1.51 % 0.04 %

6 P 1(F ) 6.10 % 1.26 % 0.84 %

7 P 1(F ) 3.95 % 0.81 % 0.55 %

8 P 1(F ) 7.15 % 0.21 % 1.21 %

14 S 2(Ca) 3.67 % 4.47 % -0.14 %

16 S 2(Ca) 27.93 % 30.86 % -0.51 %

17 S 2(Ca) 1.61 % 2.00 % -0.07 %

18 P 2(Ca) 0.96 % 0.04 % 0.16 %

19 P 2(Ca) 8.50 % 0.15 % 1.45 %

20 P 2(Ca) 0.02 % 0.60 % -0.10 %

21 P 2(Ca) 2.62 % 2.39 % 0.04 %

22 P 2(Ca) 1.22 % 0.83 % 0.07 %

23 D 2(Ca) 4.23 % 6.51 % -0.40 %

24 D 2(Ca) 10.08 % 14.36 % -0.74 %

25 D 2(Ca) 1.03 % 0.83 % 0.03 %

29 S 3(N ) 2.27 % 2.76 % -0.09 %

31 P 3(N ) 0.00 % 1.03 % -0.18 %

33 P 3(N ) 1.15 % 1.85 % -0.12 %

40 S 4(F ) 1.69 % 2.07 % -0.07 %

41 S 4(F ) 2.03 % 1.67 % 0.06 %

42 P 4(F ) 1.05 % 3.91 % -0.50 %

43 P 4(F ) 1.48 % 1.38 % 0.02 %

44 P 4(F ) 0.52 % 3.44 % -0.51 %

51 S 5(F ) 1.69 % 2.07 % -0.07 %

52 S 5(F ) 2.03 % 1.67 % 0.06 %

53 P 5(F ) 1.05 % 3.91 % -0.50 %

54 P 5(F ) 1.48 % 1.38 % 0.02 %

55 P 5(F ) 0.52 % 3.44 % -0.51 %

Contribution of various types of shells to NOCV pair/orbitals:

Type Orb. 3 Orb. 158 Pair 3

s: 46.10 % 51.11 % -0.87 %

p: 38.10 % 26.90 % 1.95 %

d: 15.81 % 21.98 % -1.07 %

f: 0.00 % 0.01 % -0.00 %

g: 0.00 % 0.00 % 0.00 %

h: 0.00 % 0.00 % 0.00 %

Contribution of each atom to NOCV pair/orbitals:

Atom Orb. 3 Orb. 158 Pair 3

1(F ): 19.15 % 4.24 % 2.60 %

2(Ca): 62.18 % 63.31 % -0.20 %

3(N ): 4.48 % 6.55 % -0.36 %

4(F ): 7.09 % 12.95 % -1.02 %

5(F ): 7.09 % 12.95 % -1.02 %


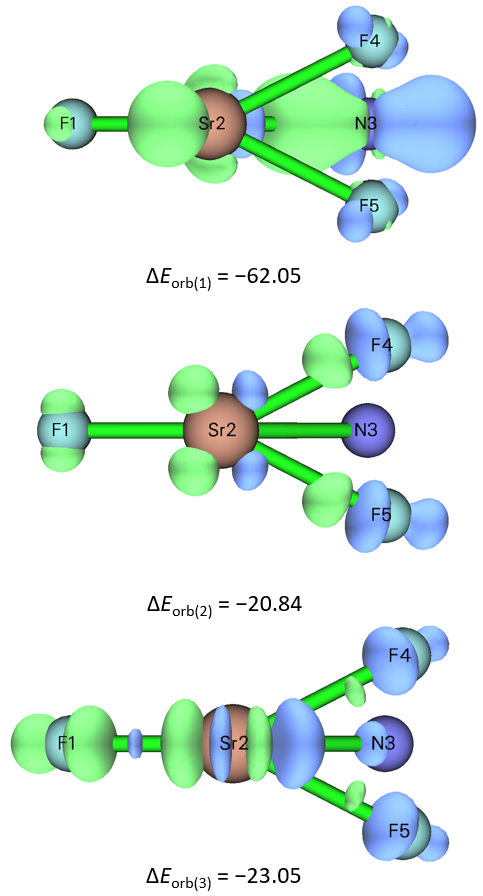


**Figure S12.** Deformation densities (the electronic charge flow is blue to green) and corresponding Δ*E*_orb_ interaction energies in kJ mol^−1^ from ETS-NOCV calculations for F'SrNF_2_ performed at B3LYP/def2-TZVP level for F'Sr^+^ and NF_2_^−^ fragments. The isosurface values is 0.002 au. The orbital interaction energies Δ*E* are given in kJ mol^−1^.

Contribution of each basis function shell to NOCV pair/orbitals:

Shell Type Atom Orb. 1 Orb. 157 Pair 1

14 S 2(Sr) 0.61 % 0.00 % 0.21 %

15 S 2(Sr) 0.26 % 0.58 % -0.11 %

16 S 2(Sr) 2.08 % 1.59 % 0.17 %

18 P 2(Sr) 0.68 % 0.93 % -0.09 %

20 P 2(Sr) 4.57 % 3.50 % 0.37 %

22 D 2(Sr) 11.82 % 9.56 % 0.78 %

23 D 2(Sr) 4.31 % 3.45 % 0.30 %

28 S 3(N ) 1.91 % 3.06 % -0.40 %

29 S 3(N ) 37.32 % 27.04 % 3.55 %

30 P 3(N ) 3.78 % 9.02 % -1.81 %

31 P 3(N ) 5.62 % 3.12 % 0.86 %

32 P 3(N ) 9.42 % 19.78 % -3.57 %

40 S 4(F ) 5.32 % 3.65 % 0.58 %

41 P 4(F ) 0.33 % 0.97 % -0.22 %

43 P 4(F ) 1.64 % 3.37 % -0.60 %

51 S 5(F ) 5.32 % 3.65 % 0.58 %

52 P 5(F ) 0.33 % 0.97 % -0.22 %

54 P 5(F ) 1.64 % 3.37 % -0.60 %

Contribution of various types of shells to NOCV pair/orbitals:

Type Orb. 1 Orb. 157 Pair 1

s: 53.50 % 40.65 % 4.44 %

p: 29.68 % 45.85 % -5.58 %

d: 16.82 % 13.50 % 1.15 %

f: 0.00 % 0.01 % -0.00 %

g: 0.00 % 0.00 % 0.00 %

h: 0.00 % 0.00 % 0.00 %

Contribution of each atom to NOCV pair/orbitals:

Atom Orb. 1 Orb. 157 Pair 1

1(F ): 0.83 % 0.04 % 0.28 %

2(Sr): 24.75 % 20.07 % 1.62 %

3(N ): 58.53 % 62.59 % -1.40 %

4(F ): 7.94 % 8.65 % -0.24 %

5(F ): 7.94 % 8.65 % -0.24 %

Contribution of each basis function shell to NOCV pair/orbitals:

Shell Type Atom Orb. 2 Orb. 156 Pair 2

6 P 1(F ) 2.54 % 0.40 % 0.35 %

7 P 1(F ) 1.21 % 0.41 % 0.13 %

8 P 1(F ) 3.26 % 0.10 % 0.52 %

18 P 2(Sr) 5.64 % 0.08 % 0.91 %

20 P 2(Sr) 1.92 % 1.25 % 0.11 %

22 D 2(Sr) 22.81 % 21.61 % 0.20 %

23 D 2(Sr) 7.65 % 7.92 % -0.04 %

31 P 3(N ) 1.03 % 0.96 % 0.01 %

39 S 4(F ) 6.65 % 7.72 % -0.18 %

40 S 4(F ) 11.07 % 7.56 % 0.57 %

41 P 4(F ) 3.17 % 7.86 % -0.77 %

42 P 4(F ) 3.36 % 3.25 % 0.02 %

43 P 4(F ) 1.92 % 6.18 % -0.70 %

50 S 5(F ) 6.65 % 7.72 % -0.18 %

51 S 5(F ) 11.07 % 7.56 % 0.57 %

52 P 5(F ) 3.17 % 7.86 % -0.77 %

53 P 5(F ) 3.36 % 3.25 % 0.02 %

54 P 5(F ) 1.92 % 6.18 % -0.70 %

Contribution of various types of shells to NOCV pair/orbitals:

Type Orb. 2 Orb. 156 Pair 2

s: 35.50 % 31.17 % 0.71 %

p: 32.65 % 38.30 % -0.93 %

d: 31.84 % 30.53 % 0.22 %

f: 0.01 % 0.01 % 0.00 %

g: 0.00 % 0.00 % 0.00 %

h: 0.00 % 0.00 % 0.00 %

Contribution of each atom to NOCV pair/orbitals:

Atom Orb. 2 Orb. 156 Pair 2

1(F ): 7.01 % 0.92 % 1.00 %

2(Sr): 38.51 % 31.60 % 1.13 %

3(N ): 1.29 % 1.13 % 0.03 %

4(F ): 26.59 % 33.17 % -1.08 %

5(F ): 26.59 % 33.17 % -1.08 %

Contribution of each basis function shell to NOCV pair/orbitals:

Shell Type Atom Orb. 3 Orb. 155 Pair 3

5 S 1(F ) 2.63 % 1.77 % 0.14 %

6 P 1(F ) 4.85 % 0.86 % 0.65 %

7 P 1(F ) 2.53 % 0.85 % 0.27 %

8 P 1(F ) 6.51 % 0.22 % 1.03 %

13 S 2(Sr) 0.68 % 0.79 % -0.02 %

14 S 2(Sr) 11.79 % 13.54 % -0.29 %

15 S 2(Sr) 1.55 % 1.77 % -0.04 %

16 S 2(Sr) 28.27 % 28.88 % -0.10 %

17 S 2(Sr) 1.49 % 1.67 % -0.03 %

18 P 2(Sr) 8.82 % 0.44 % 1.37 %

20 P 2(Sr) 2.20 % 2.37 % -0.03 %

21 P 2(Sr) 0.85 % 0.61 % 0.04 %

22 D 2(Sr) 8.54 % 12.07 % -0.58 %

23 D 2(Sr) 4.72 % 5.33 % -0.10 %

28 S 3(N ) 1.53 % 2.59 % -0.17 %

30 P 3(N ) 0.01 % 0.64 % -0.10 %

32 P 3(N ) 0.55 % 0.83 % -0.05 %

39 S 4(F ) 1.41 % 1.72 % -0.05 %

40 S 4(F ) 1.48 % 1.21 % 0.04 %

41 P 4(F ) 0.89 % 3.33 % -0.40 %

42 P 4(F ) 1.09 % 1.34 % -0.04 %

43 P 4(F ) 0.47 % 3.25 % -0.46 %

50 S 5(F ) 1.41 % 1.72 % -0.05 %

51 S 5(F ) 1.48 % 1.21 % 0.04 %

52 P 5(F ) 0.89 % 3.33 % -0.40 %

53 P 5(F ) 1.09 % 1.34 % -0.04 %

54 P 5(F ) 0.47 % 3.25 % -0.46 %

Contribution of various types of shells to NOCV pair/orbitals:

Type Orb. 3 Orb. 155 Pair 3

s: 54.62 % 58.83 % -0.69 %

p: 31.49 % 23.32 % 1.34 %

d: 13.89 % 17.85 % -0.65 %

f: 0.00 % 0.00 % -0.00 %

g: 0.00 % 0.00 % 0.00 %

h: 0.00 % 0.00 % 0.00 %

Contribution of each atom to NOCV pair/orbitals:

Atom Orb. 3 Orb. 155 Pair 3

1(F ): 16.76 % 4.04 % 2.08 %

2(Sr): 69.41 % 68.25 % 0.19 %

3(N ): 2.66 % 5.21 % -0.42 %

4(F ): 5.58 % 11.25 % -0.93 %

5(F ): 5.58 % 11.25 % -0.93 %


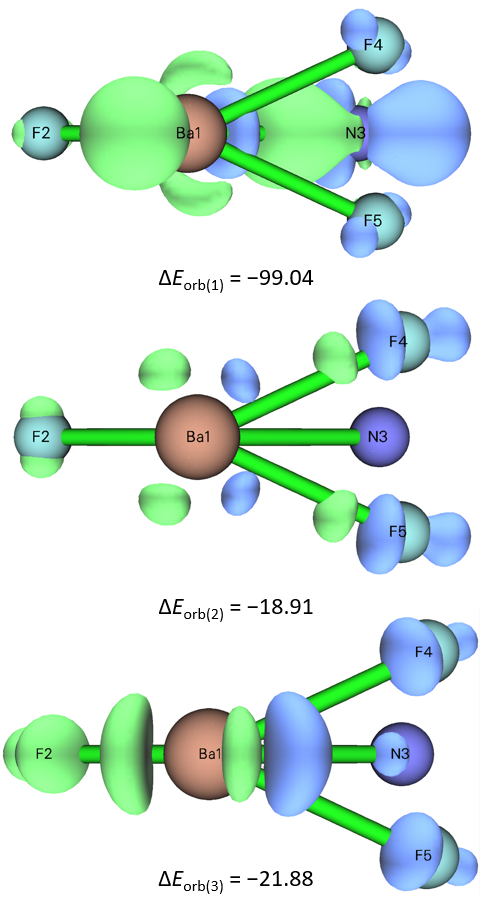


**Figure S13.** Deformation densities (the electronic charge flow is blue to green) and corresponding Δ*E*_orb_ interaction energies in kJ mol^−1^ from ETS-NOCV calculations for F'BaNF_2_ performed at B3LYP/def2-TZVP level for F'Ba^+^ and NF_2_^−^ fragments. The isosurface values is 0.002 au. The orbital interaction energies Δ*E* are given in kJ mol^−1^.

Contribution of each basis function shell to NOCV pair/orbitals:

Shell Type Atom Orb. 1 Orb. 164 Pair 1

2 S 1(Ba) 0.02 % 0.82 % -0.31 %

3 S 1(Ba) 1.00 % 0.06 % 0.36 %

5 S 1(Ba) 0.94 % 0.65 % 0.11 %

7 P 1(Ba) 0.17 % 2.67 % -0.95 %

8 P 1(Ba) 1.56 % 0.05 % 0.58 %

9 P 1(Ba) 4.10 % 2.84 % 0.48 %

10 P 1(Ba) 0.54 % 0.37 % 0.07 %

11 D 1(Ba) 16.20 % 11.69 % 1.71 %

12 D 1(Ba) 5.69 % 3.99 % 0.65 %

22 P 2(F ) 0.62 % 0.20 % 0.16 %

29 S 3(N ) 1.43 % 2.46 % -0.39 %

30 S 3(N ) 29.89 % 18.66 % 4.27 %

31 P 3(N ) 4.46 % 11.47 % -2.67 %

32 P 3(N ) 6.70 % 4.08 % 1.00 %

33 P 3(N ) 7.83 % 22.33 % -5.51 %

41 S 4(F ) 5.65 % 3.49 % 0.82 %

42 P 4(F ) 0.36 % 0.91 % -0.21 %

44 P 4(F ) 1.57 % 2.68 % -0.42 %

52 S 5(F ) 5.65 % 3.49 % 0.82 %

53 P 5(F ) 0.36 % 0.91 % -0.21 %

55 P 5(F ) 1.57 % 2.68 % -0.42 %

Contribution of various types of shells to NOCV pair/orbitals:

Type Orb. 1 Orb. 164 Pair 1

s: 45.96 % 31.58 % 5.46 %

p: 30.98 % 51.95 % -7.97 %

d: 22.57 % 16.12 % 2.45 %

f: 0.49 % 0.34 % 0.06 %

g: 0.00 % 0.00 % 0.00 %

h: 0.00 % 0.00 % 0.00 %

Contribution of each atom to NOCV pair/orbitals:

Atom Orb. 1 Orb. 164 Pair 1

1(Ba): 31.13 % 24.25 % 2.62 %

2(F ): 1.59 % 0.50 % 0.41 %

3(N ): 50.74 % 59.65 % -3.38 %

4(F ): 8.27 % 7.80 % 0.18 %

5(F ): 8.27 % 7.80 % 0.18 %

Contribution of each basis function shell to NOCV pair/orbitals:

Shell Type Atom Orb. 2 Orb. 163 Pair 2

7 P 1(Ba) 1.32 % 1.38 % -0.01 %

8 P 1(Ba) 2.42 % 0.09 % 0.37 %

9 P 1(Ba) 2.84 % 2.11 % 0.12 %

10 P 1(Ba) 0.62 % 0.48 % 0.02 %

11 D 1(Ba) 22.95 % 21.40 % 0.25 %

12 D 1(Ba) 5.04 % 5.03 % 0.00 %

14 F 1(Ba) 1.26 % 0.93 % 0.05 %

20 P 2(F ) 2.01 % 0.22 % 0.28 %

21 P 2(F ) 0.95 % 0.24 % 0.11 %

22 P 2(F ) 2.68 % 0.12 % 0.41 %

32 P 3(N ) 0.96 % 0.94 % 0.00 %

40 S 4(F ) 6.73 % 7.53 % -0.13 %

41 S 4(F ) 12.35 % 8.91 % 0.55 %

42 P 4(F ) 3.17 % 7.46 % -0.68 %

43 P 4(F ) 2.90 % 3.49 % -0.09 %

44 P 4(F ) 2.69 % 5.49 % -0.45 %

51 S 5(F ) 6.73 % 7.53 % -0.13 %

52 S 5(F ) 12.35 % 8.91 % 0.55 %

53 P 5(F ) 3.17 % 7.46 % -0.68 %

54 P 5(F ) 2.90 % 3.49 % -0.09 %

55 P 5(F ) 2.69 % 5.49 % -0.45 %

Contribution of various types of shells to NOCV pair/orbitals:

Type Orb. 2 Orb. 163 Pair 2

s: 38.22 % 33.25 % 0.79 %

p: 31.66 % 38.76 % -1.13 %

d: 28.86 % 27.05 % 0.29 %

f: 1.27 % 0.94 % 0.05 %

g: 0.00 % 0.00 % 0.00 %

h: 0.00 % 0.00 % 0.00 %

Contribution of each atom to NOCV pair/orbitals:

Atom Orb. 2 Orb. 163 Pair 2

1(Ba): 36.54 % 31.48 % 0.80 %

2(F ): 5.63 % 0.58 % 0.80 %

3(N ): 1.49 % 1.34 % 0.02 %

4(F ): 28.17 % 33.30 % -0.82 %

5(F ): 28.17 % 33.30 % -0.82 %

Contribution of each basis function shell to NOCV pair/orbitals:

Shell Type Atom Orb. 3 Orb. 162 Pair 3

2 S 1(Ba) 2.07 % 2.20 % -0.02 %

3 S 1(Ba) 17.50 % 19.39 % -0.30 %

4 S 1(Ba) 5.61 % 6.29 % -0.11 %

5 S 1(Ba) 12.31 % 12.28 % 0.00 %

6 S 1(Ba) 0.86 % 0.93 % -0.01 %

7 P 1(Ba) 2.44 % 2.30 % 0.02 %

8 P 1(Ba) 6.30 % 0.04 % 0.99 %

9 P 1(Ba) 0.43 % 0.85 % -0.07 %

10 P 1(Ba) 0.71 % 0.73 % -0.00 %

11 D 1(Ba) 11.28 % 14.95 % -0.58 %

12 D 1(Ba) 4.17 % 4.75 % -0.09 %

19 S 2(F ) 4.23 % 2.68 % 0.24 %

20 P 2(F ) 4.00 % 0.70 % 0.52 %

21 P 2(F ) 1.93 % 0.79 % 0.18 %

22 P 2(F ) 7.57 % 0.64 % 1.09 %

29 S 3(N ) 3.79 % 4.85 % -0.17 %

30 S 3(N ) 1.57 % 1.06 % 0.08 %

31 P 3(N ) 0.03 % 0.60 % -0.09 %

32 P 3(N ) 0.26 % 0.50 % -0.04 %

40 S 4(F ) 1.57 % 1.99 % -0.07 %

41 S 4(F ) 1.39 % 1.18 % 0.03 %

42 P 4(F ) 1.10 % 3.52 % -0.38 %

43 P 4(F ) 1.01 % 1.68 % -0.11 %

44 P 4(F ) 0.41 % 2.29 % -0.30 %

51 S 5(F ) 1.57 % 1.99 % -0.07 %

52 S 5(F ) 1.39 % 1.18 % 0.03 %

53 P 5(F ) 1.10 % 3.52 % -0.38 %

54 P 5(F ) 1.01 % 1.68 % -0.11 %

55 P 5(F ) 0.41 % 2.29 % -0.30 %

Contribution of various types of shells to NOCV pair/orbitals:

Type Orb. 3 Orb. 162 Pair 3

s: 54.72 % 57.37 % -0.42 %

p: 28.75 % 22.15 % 1.04 %

d: 16.16 % 20.20 % -0.64 %

f: 0.38 % 0.27 % 0.02 %

g: 0.00 % 0.00 % 0.00 %

h: 0.00 % 0.00 % 0.00 %

Contribution of each atom to NOCV pair/orbitals:

Atom Orb. 3 Orb. 162 Pair 3

1(Ba): 64.87 % 65.67 % -0.13 %

2(F ): 18.04 % 5.05 % 2.05 %

3(N ): 5.83 % 7.32 % -0.24 %

4(F ): 5.63 % 10.98 % -0.84 %

5(F ): 5.63 % 10.98 % -0.84 %

Comparison of different isomers of F'MNF_2_

**Table S1.** Relative energies of different isomers of F’MNF_2_ at B3LYP/def2-TZVP level in kJ mol^−1^.

|  | side-on *C*_s_ | end-on *C*_2v_^a^ |
| --- | --- | --- |
| Ca | 0 | 74.0 |
| Sr | 0 | 73.4 |
| Ba | 0 | 74.7 |

^a^ three imaginary vibrational wavenumbers each.

**Table S2.** Comparison between experimental and calculated vibrational wavenumbers of different isomers of F’MNF_2_ at B3LYP/def2-TZVP level in cm^−1^.

| F’CaNF_2_ | | | | F’SrNF_2_ | | | | F’BaNF_2_ | | | |
| --- | --- | --- | --- | --- | --- | --- | --- | --- | --- | --- | --- |
| In Ar | In Ne | side-on | end-on | In Ar | In Ne | side-on | end-on | In Ar | In Ne | side-on | end-on |
| − | − | 57.96 | i98.57 | − | − | 41.89 | i103.13 | − | − | 28.78 | i107.44 |
| − | − | 67.15 | i25.09 | − | − | 58.83 | i48.16 | − | − | 57.79 | i59.26 |
| − | − | 208.04 | i15.57 | − | − | 182.35 | i39.30 | − | − | 159.84 | i48.11 |
| − | − | 253.09 | 206.02 | − | − | 222.12 | 205.38 | − | − | 198.04 | 198.72 |
| − | − | 389.33 | 276.64 | − | − | 358.03 | 230.34 | − | − | 344.56 | 199.87 |
| − | − | 462.35 | 502.65 | − | − | 447.55 | 470.57 | − | − | 436.70 | 424.79 |
| 552.2 | 569.3 | 580.50 | 573.13 | 459.6 | − | 478.60 | 489.17 | − | − | 441.38 | 478.31 |
| 652.7 | 649.6 | 639.72 | 844.25 | 653.9 | 653.4 | 640.75 | 818.52 | 663.7 | 662.4 | 654.78 | 798.26 |
| 814.8 | 809.7 | 826.27 | 1072.68 | 817.2 | 812.8 | 829.89 | 1039.97 | 821.1 | 816.1 | 839.47 | 1012.87 |

QTAIM analysis data

**Table S3.** Properties of selected bond critical points (M–N/F) for side-on F'MNF_2_ at the B3LYP/def2-TZVP level. All values are in atomic units.

| bond | Electron density  *ρ* | Laplacian of electron density ∇^2^*ρ* | energy density H | ELF | Potential energy density V(r) | Lagrangian kinetic energy G(r) | \|V(r)\|/G |
| --- | --- | --- | --- | --- | --- | --- | --- |
| Ca-F' | 0.086 | 0.502 | −0.004 | 0.119 | −0.134 | 0.130 | 1.031 |
| Ca-F | 0.035 | 0.226 | 0.006 | 0.043 | −0.044 | 0.050 | 0.880 |
| Ca-N | 0.048 | 0.199 | −0.002 | 0.113 | −0.054 | 0.052 | 1.038 |
| Sr-F' | 0.076 | 0.382 | −0.007 | 0.129 | −0.110 | 0.102 | 1.078 |
| Sr-F | 0.030 | 0.178 | 0.005 | 0.042 | −0.034 | 0.040 | 0.850 |
| Sr-N | 0.044 | 0.153 | −0.003 | 0.130 | −0.044 | 0.041 | 1.073 |
| Ba-F' | 0.078 | 0.298 | −0.014 | 0.185 | −0.100 | 0.086 | 1.163 |
| Ba-F | Not detectable due to too low electron density | | |  |  |  |  |
| Ba-N | 0.044 | 0.125 | −0.004 | 0.176 | −0.038 | 0.035 | 1.086 |

**Table S4.** AIM charges for F'MNF_2_ at the B3LYP/def2-TZVP level. All values are in atomic units.

| Property | Atom | F'CaNF_2_ | F'SrNF_2_ | F'BaNF_2_ |
| --- | --- | --- | --- | --- |
| AIM Charge | F' | −0.82 | −0.84 | −0.83 |
|  | M | 1.66 | 1.69 | 1.67 |
|  | N | 0.07 | 0.07 | 0.07 |
|  | F | −0.45 | −0.46 | −0.46 |

Calculated molecular structures and vibrational data

Calculated atomic coordinates (in Å) of species for optimized structures and (an)harmonic vibrational wavenumbers (in cm^−1^) at B3LYP/def2-TZVP level.

**FCaNF_2_ (end-on, *C*_2v_)**

Ca 0.00000000 0.00000000 0.98444500

F 0.00000000 0.00000000 2.97921900

N 0.00000000 0.00000000 -1.23335900

F 0.00000000 1.11271200 -2.10379700

F 0.00000000 -1.11271200 -2.10379700

| mode | symmetry | wavenumber | IR intensity |
| --- | --- | --- | --- |
| 1 | B_2_ | -98.57 | 34.3861 |
| 2 | B_2_ | -25.09 | 42.1017 |
| 3 | B_1_ | -15.57 | 61.4683 |
| 4 | B_1_ | 206.02 | 35.3600 |
| 5 | A_1_ | 276.64 | 27.9197 |
| 6 | A_1_ | 502.65 | 3.3533 |
| 7 | A_1_ | 573.13 | 280.4784 |
| 8 | B_2_ | 844.25 | 148.3259 |
| 9 | A_1_ | 1072.68 | 19.2333 |

**FCaNF_2_ (side-on, *C*_s_)**

F -0.15905610 2.59859205 0.00000000

N -0.74961400 -1.53126396 0.00000000

F 0.23476163 -1.39409785 -1.14075802

F 0.23476163 -1.39409785 1.14075802

Ca 0.12426895 0.62740706 0.00000000

| mode | symmetry | wavenumber | IR intensity |
| --- | --- | --- | --- |
| 1 | A" | 57.96 | 55.6549 |
| 2 | A' | 67.15 | 69.3732 |
| 3 | A" | 208.04 | 3.4385 |
| 4 | A' | 253.09 | 10.6549 |
| 5 | A' | 389.33 | 21.3825 |
| 6 | A' | 462.35 | 4.4692 |
| 7 | A' | 580.50 | 244.0491 |
| 8 | A" | 639.72 | 189.3703 |
| 9 | A' | 826.27 | 49.8248 |

**Anharmonic Infrared Spectroscopy**

Units: Transition energies (E) in cm^-1

Integrated intensity (I) in km·mol^-1

Fundamental Bands

-----------------

Mode(n) E(harm) E(anharm) I(harm) I(anharm)

1(1) 826.133 795.930 49.82164046 23.02876595

2(1) 580.416 575.313 243.79435051 240.59383588

3(1) 461.998 451.719 4.48365133 4.13817803

4(1) 389.115 376.299 21.55140384 21.61138666

5(1) 252.724 243.154 10.68531578 7.73393635

6(1) 67.534 61.022 69.28376963 66.79939945

7(1) 639.539 625.710 189.46062708 169.58042998

8(1) 207.606 199.663 3.45176235 2.77965023

9(1) 58.250 54.828 55.59362414 54.90632358

Overtones

---------

Mode(n) E(harm) E(anharm) I(anharm)

1(2) 1652.266 1622.605 0.13393329

2(2) 1160.833 1146.033 1.56303617

3(2) 923.997 901.862 0.05848786

4(2) 778.230 747.354 0.58988588

5(2) 505.448 486.573 0.20877533

6(2) 135.069 120.775 0.08465051

7(2) 1279.078 1248.777 0.72177384

8(2) 415.212 395.152 1.92284095

9(2) 116.500 108.886 0.11508331

Combination Bands

-----------------

Mode(n) Mode(n) E(harm) E(anharm) I(anharm)

2(1) 1(1) 1406.549 1387.783 0.01230862

3(1) 1(1) 1288.132 1262.583 0.23440199

3(1) 2(1) 1042.415 1026.672 0.03086813

4(1) 1(1) 1215.248 1188.165 0.24682094

4(1) 2(1) 969.531 950.712 0.51120612

4(1) 3(1) 851.113 825.500 1.83554021

5(1) 1(1) 1078.857 1056.480 0.26679784

5(1) 2(1) 833.141 820.317 13.28973120

5(1) 3(1) 714.723 690.784 0.58209284

5(1) 4(1) 641.839 615.930 0.03484390

6(1) 1(1) 893.667 876.468 0.20241672

6(1) 2(1) 647.951 635.776 0.23729674

6(1) 3(1) 529.533 511.871 0.01691925

6(1) 4(1) 456.649 436.363 0.33894765

6(1) 5(1) 320.259 303.963 0.13939202

7(1) 1(1) 1465.672 1427.961 2.52543732

7(1) 2(1) 1219.956 1201.133 0.01401989

7(1) 3(1) 1101.538 1069.614 0.00055041

7(1) 4(1) 1028.654 999.794 0.01009187

7(1) 5(1) 892.263 869.258 0.20722142

7(1) 6(1) 707.073 688.051 0.06156891

8(1) 1(1) 1033.739 1016.085 0.60372900

8(1) 2(1) 788.023 775.388 0.34917034

8(1) 3(1) 669.605 654.125 13.02891868

8(1) 4(1) 596.721 573.353 1.46618915

8(1) 5(1) 460.330 432.623 0.32804482

8(1) 6(1) 275.140 259.289 0.00093824

8(1) 7(1) 847.145 841.915 14.87393140

9(1) 1(1) 884.383 871.554 0.00074102

9(1) 2(1) 638.667 630.562 10.65924821

9(1) 3(1) 520.249 505.446 0.00329958

9(1) 4(1) 447.365 431.096 0.03041466

9(1) 5(1) 310.974 296.202 0.10940856

9(1) 6(1) 125.785 106.856 0.00236686

9(1) 7(1) 697.789 682.734 0.45211411

9(1) 8(1) 265.856 256.220 2.45284470

**FCa^15^NF_2_ (side-on, *C*_s_)**

| mode | symmetry | wavenumber | IR intensity |
| --- | --- | --- | --- |
| 1 | A" | 58.07 | 55.6051 |
| 2 | A' | 67.61 | 68.7830 |
| 3 | A" | 207.00 | 3.2912 |
| 4 | A' | 252.14 | 10.3301 |
| 5 | A' | 381.72 | 22.8231 |
| 6 | A' | 458.16 | 3.7041 |
| 7 | A' | 579.94 | 242.8220 |
| 8 | A" | 628.39 | 184.7808 |
| 9 | A' | 808.72 | 49.3604 |
|  |  |  |  |

**Anharmonic Infrared Spectroscopy**

Fundamental Bands

-----------------

Mode(n) E(harm) E(anharm) I(harm) I(anharm)

1(1) 808.409 780.300 49.39320570 31.13007838

2(1) 580.190 575.395 242.64912583 239.49259454

3(1) 458.271 449.887 3.69783179 3.12330213

4(1) 381.980 372.071 22.91406324 22.72971901

5(1) 252.297 243.406 10.37680195 7.87141838

6(1) 67.270 69.573 68.70006529 69.35299097

7(1) 627.935 613.364 184.64963492 171.38845279

8(1) 206.969 201.141 3.27360372 2.58860043

9(1) 58.246 61.580 55.56408053 55.70058933

Overtones

---------

Mode(n) E(harm) E(anharm) I(anharm)

1(2) 1616.818 1585.502 0.13114354

2(2) 1160.381 1146.179 1.58071475

3(2) 916.543 898.409 0.05194284

4(2) 763.961 738.838 0.67121706

5(2) 504.595 487.430 0.17900734

6(2) 134.539 139.458 0.09973266

7(2) 1255.870 1223.694 0.67528788

8(2) 413.939 397.955 1.48903016

9(2) 116.492 124.237 0.13217279

Combination Bands

-----------------

Mode(n) Mode(n) E(harm) E(anharm) I(anharm)

2(1) 1(1) 1388.599 1370.274 0.01052261

3(1) 1(1) 1266.680 1242.254 0.20520152

3(1) 2(1) 1038.462 1025.063 0.01718701

4(1) 1(1) 1190.389 1165.499 0.23392531

4(1) 2(1) 962.171 946.657 0.50863250

4(1) 3(1) 840.252 819.640 1.02555665

5(1) 1(1) 1060.706 1038.989 0.25866572

5(1) 2(1) 832.488 819.592 1.60130374

5(1) 3(1) 710.569 689.479 0.60404365

5(1) 4(1) 634.278 612.307 0.04055278

6(1) 1(1) 875.679 864.794 0.19182144

6(1) 2(1) 647.460 644.706 0.25050925

6(1) 3(1) 525.541 519.840 0.00719640

6(1) 4(1) 449.250 442.671 0.63626051

6(1) 5(1) 319.567 312.698 0.14688898

7(1) 1(1) 1436.344 1396.952 2.36995652

7(1) 2(1) 1208.125 1188.464 0.01342522

7(1) 3(1) 1086.206 1055.924 0.00004607

7(1) 4(1) 1009.915 983.181 0.01018645

7(1) 5(1) 880.232 856.713 0.18804939

7(1) 6(1) 695.205 682.732 0.05729119

8(1) 1(1) 1015.378 998.582 0.59411669

8(1) 2(1) 787.160 776.991 0.29831673

8(1) 3(1) 665.241 653.394 6.82915236

8(1) 4(1) 588.950 571.525 2.18330078

8(1) 5(1) 459.267 434.889 0.37827645

8(1) 6(1) 274.239 272.710 0.00111824

8(1) 7(1) 834.904 828.266 14.25778798

9(1) 1(1) 866.655 857.302 0.00071342

9(1) 2(1) 638.436 637.501 3.40241192

9(1) 3(1) 516.517 511.421 0.00356742

9(1) 4(1) 440.226 435.657 0.03063070

9(1) 5(1) 310.543 303.207 0.10934209

9(1) 6(1) 125.516 132.693 0.00286256

9(1) 7(1) 686.181 675.231 0.46870790

9(1) 8(1) 265.215 264.408 2.46441300

**FSrNF_2_ (end-on, *C*_2v_)**

F 0.00000000 0.00000000 2.92268400

N 0.00000000 0.00000000 -1.60528600

F 0.00000000 1.11636300 -2.48506700

F 0.00000000 -1.11636300 -2.48506700

Sr 0.00000000 0.00000000 0.78063300

| mode | symmetry | wavenumber | IR intensity |
| --- | --- | --- | --- |
| 1 | B_2_ | -103.13 | 22.0583 |
| 2 | B_2_ | -48.16 | 48.5443 |
| 3 | B_1_ | -39.30 | 31.9336 |
| 4 | B_1_ | 205.38 | 22.5879 |
| 5 | A_1_ | 230.34 | 37.0603 |
| 6 | A_1_ | 470.57 | 183.6263 |
| 7 | A_1_ | 489.17 | 10.8765 |
| 8 | B_2_ | 818.52 | 154.2371 |
| 9 | A_1_ | 1039.97 | 36.6351 |

**FSrNF_2_ (side-on, *C*_s_)**

F -0.44427577 -2.55270517 0.00000000

N -0.85098185 1.72513582 0.00000000

F 0.13490480 1.70305782 1.14233195

F 0.13490480 1.70305782 -1.14233195

Sr 0.19982919 -0.52170816 0.00000000

| mode | symmetry | wavenumber | IR intensity |
| --- | --- | --- | --- |
| 1 | A" | 41.89 | 38.9891 |
| 2 | A' | 58.83 | 44.7187 |
| 3 | A" | 182.35 | 0.8516 |
| 4 | A' | 222.12 | 18.8109 |
| 5 | A' | 358.03 | 29.4004 |
| 6 | A' | 447.55 | 3.4921 |
| 7 | A' | 478.60 | 151.0965 |
| 8 | A" | 640.75 | 181.7763 |
| 9 | A' | 829.89 | 46.0161 |

**Anharmonic Infrared Spectroscopy**

Units: Transition energies (E) in cm^-1

Integrated intensity (I) in km·mol^-1

Fundamental Bands

-----------------

Mode(n) E(harm) E(anharm) I(harm) I(anharm)

1(1) 829.956 832.178 46.01172360 28.40461427

2(1) 478.598 474.480 151.13548475 150.64929214

3(1) 447.610 444.147 3.50013943 1.63395440

4(1) 358.126 345.869 29.29590400 24.51644677

5(1) 221.815 221.405 18.87349305 10.83113669

6(1) 58.878 55.309 44.76152663 44.80586018

7(1) 640.844 630.308 181.70930438 133.83575769

8(1) 181.840 177.295 0.86500301 0.77484807

9(1) 41.894 38.928 39.02455361 38.84747101

Overtones

---------

Mode(n) E(harm) E(anharm) I(anharm)

1(2) 1659.913 1631.854 0.14140734

2(2) 957.195 944.971 1.21687452

3(2) 895.219 877.700 0.01595914

4(2) 716.253 685.821 0.42425550

5(2) 443.631 429.154 1.05024838

6(2) 117.756 110.046 0.08463566

7(2) 1281.688 1247.493 0.69487368

8(2) 363.680 352.260 6.89452240

9(2) 83.787 78.625 0.12114031

Combination Bands

-----------------

Mode(n) Mode(n) E(harm) E(anharm) I(anharm)

2(1) 1(1) 1308.554 1293.148 0.00541458

3(1) 1(1) 1277.566 1257.092 0.19051456

3(1) 2(1) 926.207 913.982 0.01940163

4(1) 1(1) 1188.083 1165.412 0.34736765

4(1) 2(1) 836.724 820.652 0.76514146

4(1) 3(1) 805.736 781.449 3.09968785

5(1) 1(1) 1051.772 1036.229 0.28189889

5(1) 2(1) 700.413 691.234 0.02040087

5(1) 3(1) 669.425 655.343 0.08123665

5(1) 4(1) 579.942 561.051 0.02109021

6(1) 1(1) 888.834 873.992 0.11261755

6(1) 2(1) 537.476 529.669 0.28442926

6(1) 3(1) 506.487 494.881 0.00975014

6(1) 4(1) 417.004 400.737 0.28959170

6(1) 5(1) 280.693 272.022 0.02671640

7(1) 1(1) 1470.800 1431.186 2.56664762

7(1) 2(1) 1119.442 1099.653 0.00174666

7(1) 3(1) 1088.454 1058.466 0.00015201

7(1) 4(1) 998.970 968.519 0.00053136

7(1) 5(1) 862.659 842.173 0.28194657

7(1) 6(1) 699.722 680.497 0.06043702

8(1) 1(1) 1011.796 997.980 0.53023705

8(1) 2(1) 660.438 652.002 3.44069678

8(1) 3(1) 629.449 610.801 43.41497342

8(1) 4(1) 539.966 522.605 0.18910360

8(1) 5(1) 403.655 387.515 0.08834944

8(1) 6(1) 240.718 233.825 0.00080071

8(1) 7(1) 822.684 789.072 12.50259802

9(1) 1(1) 871.850 858.422 0.00001707

9(1) 2(1) 520.491 513.393 0.26428824

9(1) 3(1) 489.503 478.388 0.03077123

9(1) 4(1) 400.020 384.099 0.00098011

9(1) 5(1) 263.709 255.256 0.00523731

9(1) 6(1) 100.772 88.172 0.00532122

9(1) 7(1) 682.738 664.809 0.29394728

9(1) 8(1) 223.734 210.386 6.70697583

**FSr^15^NF_2_ (side-on, *C*_s_)**

| mode | symmetry | wavenumber | IR intensity |
| --- | --- | --- | --- |
| 1 | A" | 41.54 | 38.9979 |
| 2 | A' | 58.78 | 44.3500 |
| 3 | A" | 181.63 | 0.8054 |
| 4 | A' | 222.22 | 18.7873 |
| 5 | A' | 349.30 | 29.0661 |
| 6 | A' | 444.70 | 3.0461 |
| 7 | A' | 478.52 | 150.6911 |
| 8 | A" | 628.81 | 176.8036 |
| 9 | A' | 812.35 | 45.5721 |

**Anharmonic Infrared Spectroscopy**

Units: Transition energies (E) in cm^-1

Integrated intensity (I) in km·mol^-1

Fundamental Bands

-----------------

Mode(n) E(harm) E(anharm) I(harm) I(anharm)

1(1) 812.203 816.888 45.56502042 25.34742788

2(1) 478.565 474.387 150.78003604 150.22624301

3(1) 444.689 442.776 3.06784318 1.35868535

4(1) 349.339 337.956 29.04016353 28.34575634

5(1) 221.755 221.154 18.67447859 11.15168047

6(1) 58.693 55.411 44.45747299 44.50117309

7(1) 628.810 621.298 176.81567140 97.55341946

8(1) 181.388 176.766 0.79342100 0.71076928

9(1) 41.893 38.839 39.04224245 38.84364056

Overtones

---------

Mode(n) E(harm) E(anharm) I(anharm)

1(2) 1624.405 1598.246 0.13859671

2(2) 957.130 944.771 1.21951142

3(2) 889.378 872.640 0.01823134

4(2) 698.678 670.120 0.41806423

5(2) 443.510 428.138 1.25379273

6(2) 117.386 110.297 0.08109878

7(2) 1257.621 1225.396 0.65515846

8(2) 362.775 350.591 2.39664307

9(2) 83.786 78.442 0.12048014

Combination Bands

-----------------

Mode(n) Mode(n) E(harm) E(anharm) I(anharm)

2(1) 1(1) 1290.768 1276.199 0.00448007

3(1) 1(1) 1256.892 1237.830 0.16896043

3(1) 2(1) 923.254 911.232 0.01456031

4(1) 1(1) 1161.542 1140.924 0.32585793

4(1) 2(1) 827.904 812.600 0.12923822

4(1) 3(1) 794.028 771.219 4.57785135

5(1) 1(1) 1033.958 1019.270 0.27601143

5(1) 2(1) 700.320 691.136 0.02114333

5(1) 3(1) 666.444 652.818 0.08351998

5(1) 4(1) 571.094 553.203 0.02076591

6(1) 1(1) 870.896 857.275 0.10704346

6(1) 2(1) 537.258 529.645 0.29082247

6(1) 3(1) 503.382 492.746 0.00781443

6(1) 4(1) 408.032 392.839 0.27130903

6(1) 5(1) 280.448 272.225 0.02648721

7(1) 1(1) 1441.013 1403.569 2.41562361

7(1) 2(1) 1107.375 1088.558 0.00159797

7(1) 3(1) 1073.499 1044.912 0.00012272

7(1) 4(1) 978.149 949.697 0.00057466

7(1) 5(1) 850.565 831.002 0.26070930

7(1) 6(1) 687.503 669.639 0.05631009

8(1) 1(1) 993.590 980.405 0.52077245

8(1) 2(1) 659.953 651.229 1.24161626

8(1) 3(1) 626.077 605.542 76.91728220

8(1) 4(1) 530.727 514.865 0.21953487

8(1) 5(1) 403.143 386.993 0.09908959

8(1) 6(1) 240.081 233.452 0.00086747

8(1) 7(1) 810.198 775.799 14.36734977

9(1) 1(1) 854.096 841.393 0.00002613

9(1) 2(1) 520.458 513.215 0.26530777

9(1) 3(1) 486.582 475.765 0.04117435

9(1) 4(1) 391.232 376.069 0.00055680

9(1) 5(1) 263.648 255.154 0.00506098

9(1) 6(1) 100.586 88.000 0.00523922

9(1) 7(1) 670.704 653.546 0.28789394

9(1) 8(1) 223.281 210.062 6.18940004

**FBaNF_2_ (end-on, *C*_2v_)**

F 0.00000000 0.00000000 2.92223400

N 0.00000000 0.00000000 -1.89599800

F 0.00000000 1.11937000 -2.78289500

F 0.00000000 -1.11937000 -2.78289500

Ba 0.00000000 0.00000000 0.66185700

| mode | symmetry | wavenumber | IR intensity |
| --- | --- | --- | --- |
| 1 | B_2_ | -107.44 | 16.357 |
| 2 | B_2_ | -59.26 | 35.8287 |
| 3 | B_1_ | -48.11 | 25.0776 |
| 4 | B_1_ | 198.72 | 25.0371 |
| 5 | A_1_ | 199.87 | 45.8221 |
| 6 | A_1_ | 424.79 | 191.824 |
| 7 | A_1_ | 478.31 | 9.4761 |
| 8 | B_2_ | 798.26 | 162.9103 |
| 9 | A_1_ | 1012.87 | 53.9768 |

**FBaNF_2_ (side-on, *C*_s_)**

Ba -0.33204400 0.41612900 0.00000000

F 0.35518300 2.54658800 0.00000000

N 1.28636300 -1.65218100 0.00000000

F 0.35518300 -1.92540300 1.13860000

F 0.35518300 -1.92540300 -1.13860000

| mode | symmetry | wavenumber | IR intensity |
| --- | --- | --- | --- |
| 1 | A" | 28.78 | 23.1045 |
| 2 | A' | 57.79 | 28.7484 |
| 3 | A" | 159.84 | 0.7715 |
| 4 | A' | 198.04 | 22.7682 |
| 5 | A' | 344.56 | 43.1297 |
| 6 | A' | 436.70 | 101.6005 |
| 7 | A' | 441.38 | 43.0179 |
| 8 | A" | 654.78 | 173.0794 |
| 9 | A' | 839.47 | 44.4530 |

**Anharmonic Infrared Spectroscopy**

Units: Transition energies (E) in cm^-1

Integrated intensity (I) in km·mol^-1

Fundamental Bands

-----------------

Mode(n) E(harm) E(anharm) I(harm) I(anharm)

1(1) 839.262 838.515 44.40968929 35.64630910

2(1) 441.228 434.129 44.87670329 189.45215638

3(1) 436.593 431.373 99.85047449 29.61387271

4(1) 344.491 331.553 43.06283527 42.71776716

5(1) 198.266 193.118 22.76746535 18.84750364

6(1) 57.702 55.638 28.80357845 28.60717610

7(1) 654.473 643.320 173.13808764 169.54741238

8(1) 160.232 153.093 0.76373553 0.90454498

9(1) 28.999 19.965 23.17952671 20.33502126

Overtones

---------

Mode(n) E(harm) E(anharm) I(anharm)

1(2) 1678.525 1654.924 0.24148371

2(2) 882.456 867.288 0.05591595

3(2) 873.186 861.074 0.41612132

4(2) 688.983 656.085 0.71538179

5(2) 396.533 379.402 0.70150614

6(2) 115.404 111.906 0.11565585

7(2) 1308.946 1283.185 0.64254704

8(2) 320.465 304.020 0.93212870

9(2) 57.998 35.136 0.04816498

Combination Bands

-----------------

Mode(n) Mode(n) E(harm) E(anharm) I(anharm)

2(1) 1(1) 1280.490 1263.074 0.09014327

3(1) 1(1) 1275.855 1261.020 0.04337161

3(1) 2(1) 877.821 863.224 0.83687325

4(1) 1(1) 1183.754 1162.750 0.45361540

4(1) 2(1) 785.719 763.937 0.57081194

4(1) 3(1) 781.084 761.984 0.38707305

5(1) 1(1) 1037.529 1022.450 0.39776055

5(1) 2(1) 639.494 625.271 0.02811508

5(1) 3(1) 634.859 622.851 0.00771034

5(1) 4(1) 542.758 521.426 0.04135948

6(1) 1(1) 896.964 886.884 0.05573845

6(1) 2(1) 498.930 489.453 0.14315945

6(1) 3(1) 494.295 486.862 0.17299261

6(1) 4(1) 402.193 386.456 0.06994220

6(1) 5(1) 255.968 246.150 0.00179975

7(1) 1(1) 1493.735 1460.529 2.49913850

7(1) 2(1) 1095.701 1074.058 0.00002306

7(1) 3(1) 1091.066 1072.786 0.00018544

7(1) 4(1) 998.965 972.180 0.02342184

7(1) 5(1) 852.739 834.990 0.32587249

7(1) 6(1) 712.175 699.707 0.00099702

8(1) 1(1) 999.495 985.476 0.50815710

8(1) 2(1) 601.460 584.960 3.25612006

8(1) 3(1) 596.825 583.644 0.90705985

8(1) 4(1) 504.724 484.480 0.09274460

8(1) 5(1) 358.499 335.868 0.01279136

8(1) 6(1) 217.934 208.554 0.00141779

8(1) 7(1) 814.705 789.844 6.66866540

9(1) 1(1) 868.261 853.840 0.00286922

9(1) 2(1) 470.227 453.125 0.27867447

9(1) 3(1) 465.592 450.605 0.06224016

9(1) 4(1) 373.491 346.279 0.00116287

9(1) 5(1) 227.265 210.291 0.00211490

9(1) 6(1) 86.701 70.470 0.00997913

9(1) 7(1) 683.472 666.001 0.47174227

9(1) 8(1) 189.232 169.094 1.20355785

**FBa^15^NF_2_ (side-on, *C*_s_)**

| mode | symmetry | wavenumber | IR intensity |
| --- | --- | --- | --- |
| 1 | A" | 27.16 | 23.2070 |
| 2 | A' | 57.50 | 28.6036 |
| 3 | A" | 158.54 | 0.7295 |
| 4 | A' | 197.43 | 22.5865 |
| 5 | A' | 334.78 | 41.0307 |
| 6 | A' | 435.24 | 62.4275 |
| 7 | A' | 440.10 | 82.4569 |
| 8 | A" | 642.21 | 168.3969 |
| 9 | A' | 821.61 | 43.9051 |

**Anharmonic Infrared Spectroscopy**

Units: Transition energies (E) in cm^-1

Integrated intensity (I) in km·mol^-1

Fundamental Bands

-----------------

Mode(n) E(harm) E(anharm) I(harm) I(anharm)

1(1) 821.486 821.983 43.88493863 34.01145144

2(1) 440.098 434.282 79.58963700 193.12876654

3(1) 435.274 429.023 65.40016759 7.80829241

4(1) 334.956 322.625 40.84345618 41.04428670

5(1) 198.265 193.191 22.73250237 18.76527462

6(1) 57.540 55.574 28.62660371 28.37605417

7(1) 641.919 631.575 168.40418055 163.44590041

8(1) 159.923 152.739 0.70995817 0.84580637

9(1) 28.992 20.377 23.28567228 20.60244002

Overtones

---------

Mode(n) E(harm) E(anharm) I(anharm)

1(2) 1642.971 1620.398 0.23248637

2(2) 880.197 867.061 0.33680785

3(2) 870.548 857.069 0.09281249

4(2) 669.911 638.507 0.67081398

5(2) 396.529 379.411 0.52090272

6(2) 115.080 111.771 0.11635747

7(2) 1283.838 1259.916 0.60653170

8(2) 319.846 303.326 0.93597338

9(2) 57.983 36.034 0.03503059

Combination Bands

-----------------

Mode(n) Mode(n) E(harm) E(anharm) I(anharm)

2(1) 1(1) 1261.584 1246.388 0.05153312

3(1) 1(1) 1256.760 1240.593 0.06949671

3(1) 2(1) 875.373 860.855 0.85019989

4(1) 1(1) 1156.441 1136.342 0.42375050

4(1) 2(1) 775.054 756.076 0.34636703

4(1) 3(1) 770.230 750.189 0.59510009

5(1) 1(1) 1019.750 1005.105 0.38709729

5(1) 2(1) 638.363 625.563 0.02141823

5(1) 3(1) 633.539 620.769 0.01443877

5(1) 4(1) 533.220 512.683 0.03957134

6(1) 1(1) 879.026 869.384 0.05328868

6(1) 2(1) 497.638 489.503 0.21030300

6(1) 3(1) 492.814 484.493 0.12687133

6(1) 4(1) 392.495 377.457 0.09849401

6(1) 5(1) 255.804 246.174 0.00244900

7(1) 1(1) 1463.405 1431.853 2.34931330

7(1) 2(1) 1082.017 1064.065 0.00001872

7(1) 3(1) 1077.193 1057.329 0.00069377

7(1) 4(1) 976.874 951.716 0.02326555

7(1) 5(1) 840.183 823.311 0.30215247

7(1) 6(1) 699.459 687.864 0.00093619

8(1) 1(1) 981.409 967.701 0.49638164

8(1) 2(1) 600.021 585.457 3.13726048

8(1) 3(1) 595.197 580.136 2.33685222

8(1) 4(1) 494.878 475.135 0.09810677

8(1) 5(1) 358.187 335.587 0.01482559

8(1) 6(1) 217.463 208.139 0.00148369

8(1) 7(1) 801.842 776.994 7.97779693

9(1) 1(1) 850.477 836.945 0.00302876

9(1) 2(1) 469.090 453.713 0.31634450

9(1) 3(1) 464.266 448.520 0.00909934

9(1) 4(1) 363.947 337.878 0.00136778

9(1) 5(1) 227.256 210.931 0.00191303

9(1) 6(1) 86.531 71.098 0.01000807

9(1) 7(1) 670.910 654.873 0.45091091

9(1) 8(1) 188.914 169.138 1.23041460

Calculated atomic coordinates (in Å) of species for optimized structures and harmonic vibrational wavenumbers (in cm^−1^) at CCSD(T)/aug-cc-pwCVTZ-(PP) level.

**FCaNF_2_ (side-on, *C*_s_)**

F -0.0506556027 2.6049640540 0.0000000000

N 0.8179418923 -1.5529688283 0.0000000000

F -0.1694981664 -1.3765292496 1.1317251593

F -0.1694981664 -1.3765292496 -1.1317251593

Ca -0.0507399568 0.6129932735 0.0000000000

| symmetry | wavenumber |
| --- | --- |
| A" | 57.88 |
| A' | 61.29 |
| A" | 231.25 |
| A' | 265.42 |
| A' | 380.83 |
| A' | 466.8 |
| A' | 583.77 |
| A" | 664.78 |
| A' | 827.62 |

**FCa^15^NF_2_ (side-on, *C*_s_)**

| symmetry | wavenumber |
| --- | --- |
| A" | 57.86 |
| A' | 61.03 |
| A" | 230.64 |
| A' | 264.9 |
| A' | 373.7 |
| A' | 463.44 |
| A' | 583.52 |
| A" | 652.6 |
| A' | 809.73 |

**FSrNF_2_ (side-on, *C*_s_)**

F 0.0240720833 2.6139801782 0.0000000000

N 1.0734575798 -1.6693709420 0.0000000000

F 0.0796358099 -1.6840664873 1.1334985405

F 0.0796358099 -1.6840664873 -1.1334985405

Sr -0.1553112828 0.4899937383 0.0000000000

| symmetry | wavenumber |
| --- | --- |
| A" | 35.35 |
| A' | 45.33 |
| A" | 204.97 |
| A' | 237.92 |
| A' | 354.26 |
| A' | 454.68 |
| A' | 480.01 |
| A" | 666.24 |
| A' | 831.12 |

**FSr^15^NF_2_ (side-on, *C*_s_)**

| symmetry | wavenumber |
| --- | --- |
| A" | 35.34 |
| A' | 45.12 |
| A" | 204.52 |
| A' | 237.85 |
| A' | 345.72 |
| A' | 451.75 |
| A' | 479.96 |
| A" | 653.64 |
| A' | 813.21 |

**FBaNF_2_ (side-on, *C*_s_)**

F 0.3280362373 2.5530762633 0.0000000000

N 1.2959493970 -1.6802011489 0.0000000000

F 0.3423235504 -1.9078812688 1.1335809899

F 0.3423235504 -1.9078812688 -1.1335809899

Ba -0.2887727351 0.4026274232 0.0000000000

| symmetry | wavenumber |
| --- | --- |
| A" | 24.36 |
| A' | 52.14 |
| A" | 179.92 |
| A' | 213.87 |
| A' | 342.51 |
| A' | 433.08 |
| A' | 444.15 |
| A" | 678.97 |
| A' | 837.80 |

**FBa^15^NF_2_ (side-on, *C*_s_)**

| symmetry | wavenumber |
| --- | --- |
| A" | 24.39 |
| A' | 51.93 |
| A" | 179.59 |
| A' | 213.87 |
| A' | 333.2 |
| A' | 432.8 |
| A' | 441.85 |
| A" | 665.89 |
| A' | 820.02 |

# References

[1] M. J. Frisch, G. W. Trucks, H. B. Schlegel, G. E. Scuseria, M. A. Robb, J. R. Cheeseman, G. Scalmani, V. Barone, G. A. Petersson, H. Nakatsuji, X. Li, M. Caricato, A. V. Marenich, J. Bloino, B. G. Janesko, R. Gomperts, B. Mennucci, H. P. Hratchian, J. V. Ortiz, A. F. Izmaylov, J. L. Sonnenberg, D. Williams-Young, F. Ding, F. Lipparini, F. Egidi, J. Goings, B. Peng, A. Petrone, T. Henderson, D. Ranasinghe, V. G. Zakrzewski, J. Gao, N. Rega, G. Zheng, W. Liang, M. Hada, M. Ehara, K. Toyota, R. Fukuda, J. Hasegawa, M. Ishida, T. Nakajima, Y. Honda, O. Kitao, H. Nakai, T. Vreven, K. Throssell, J. A. Montgomery, Jr., J. E. Peralta, F. Ogliaro, M. J. Bearpark, J. J. Heyd, E. N. Brothers, K. N. Kudin, V. N. Staroverov, T. A. Keith, R. Kobayashi, J. Normand, K. Raghavachari, A. P. Rendell, J. C. Burant, S. S. Iyengar, J. Tomasi, M. Cossi, J. M. Millam, M. Klene, C. Adamo, R. Cammi, J. W. Ochterski, R. L. Martin, K. Morokuma, O. Farkas, J. B. Foresman, and D. J. Fox, *Gaussian16*, Gaussian, Inc., Wallingford CT, **2016**.

[2] P. J. Stephens, F. J. Devlin, C. F. Chabalowski, M. J. Frisch, *J. Phys. Chem.* **1994**, *98*, 11623.

[3] A. D. Becke, *J. Chem. Phys.* **1993**, *98*, 5648.

[4] C. Lee, W. Yang, R. G. Parr, *Phys. Rev. B* **1988**, *37*, 785.

[5] S. H. Vosko, L. Wilk, M. Nusair, *Can. J. Phys.* **1980**, *58*, 1200.

[6] C. Lee, W. Yang, R. G. Parr, *Phys. Rev. B* **1988**, *37*, 785.

[7] A. D. Becke, *J. Chem. Phys.* **1992**, *96*, 2155.

[8] F. Weigend, R. Ahlrichs, *Phys. Chem. Chem. Phys.* **2005**, *7*, 3297.

[9] M. Kaupp, P. v. R. Schleyer, H. Stoll, H. Preuss, *J. Chem. Phys.* **1991**, *94*, 1360.

[10] E. D. Glendening, J. K. Badenhoop, A. E. Reed, J. E. Carpenter, J. A. Bohmann, C. M. Morales, P. Karafiloglou, C. R. Landis, F. Weinhold, *NBO 7.0*, Theoretical Chemistry Institute, University of Wisconsin, Madison, WI, **2018**.

[11] T. Lu, F. Chen, *J. Comput. Chem.* **2012**, *33*, 580.

[12] H.-J. Werner, P. J. Knowles, P. Celani, W. Györffy, A. Hesselmann, D. Kats, G. Knizia, A. Köhn, T. Korona, D. Kreplin, R. Lindh, Q. Ma, F. R. Manby, A. Mitrushenkov, G. Rauhut, M. Schütz, K. R. Shamasundar, T. B. Adler, R. D. Amos, J. Baker, S. J. Bennie, A. Bernhardsson, A. Berning, J. A. Black, P. J. Bygrave, R. Cimiraglia, D. L. Cooper, D. Coughtrie, M. J. O. Deegan, A. J. Dobbyn, K. Doll and M. Dornbach, F. Eckert, S. Erfort, E. Goll, C. Hampel, G. Hetzer, J. G. Hill, M. Hodges and T. Hrenar, G. Jansen, C. Köppl, C. Kollmar, S. J. R. Lee, Y. Liu, A. W. Lloyd, R. A. Mata, A. J. May, B. Mussard, S. J. McNicholas, W. Meyer, T. F. Miller III, M. E. Mura, A. Nicklass, D. P. O'Neill, P. Palmieri, D. Peng, K. A. Peterson, K. Pflüger, R. Pitzer, I. Polyak, P. Pulay, M. Reiher, J. O. Richardson, J. B. Robinson, B. Schröder, M. Schwilk and T. Shiozaki, M. Sibaev, H. Stoll, A. J. Stone, R. Tarroni, T. Thorsteinsson, J. Toulouse, M. Wang, M. Welborn and B. Ziegler, *MOLPRO, version 2021.3, a package of ab initio programs*, **2021**.

[13] H.-J. Werner, P. J. Knowles, F. R. Manby, J. A. Black, K. Doll, A. Heßelmann, D. Kats, A. Köhn, T. Korona, D. A. Kreplin et al., *J. Chem. Phys.* **2020**, *152*, 144107.

[14] H.‐J. Werner, P. J. Knowles, G. Knizia, F. R. Manby, M. Schütz, *WIREs Comput Mol Sci* **2012**, *2*, 242.

[15] T. H. Dunning, *J. Chem. Phys.* **1989**, *90*, 1007.

[16] R. A. Kendall, T. H. Dunning, R. J. Harrison, *J. Chem. Phys.* **1992**, *96*, 6796.

[17] R. A. Kendall, T. H. Dunning, R. J. Harrison, *J. Chem. Phys.* **1992**, *96*, 6796.

[18] J. G. Hill, K. A. Peterson, *J. Chem. Phys.* **2017**, *147*, 244106.

[19] I. S. Lim, H. Stoll, P. Schwerdtfeger, *J. Chem. Phys.* **2006**, *124*, 34107.

# Author Contributions

Xiya Xia did all the experiments and calculations, wrote the initial draft of the manuscript. Robert Medel and Sebastian Riedel managed the project and revised the manuscript.
